# Performance Bounds of Ranging Precision in SPAD-Based dToF LiDAR

**DOI:** 10.3390/s25196184

**Published:** 2025-10-06

**Authors:** Hao Wu, Yingyu Wang, Shiyi Sun, Lijie Zhao, Limin Tong, Linjie Shen, Jiang Zhu

**Affiliations:** 1 Hikvision Research Institute, Hangzhou 310051, China; 2 State Key Laboratory of Extreme Photonics and Instrumentation, College of Optical Science and Engineering, Zhejiang University, Hangzhou 310027, China

**Keywords:** direct time-of-flight, single-photon avalanche diode, Cramér–Rao lower bound

## Abstract

LiDAR with direct time-of-flight (dToF) technology based on single-photon avalanche diode detectors (SPADs) has been widely adopted in various applications. However, a comprehensive theoretical understanding of its fundamental ranging performance bounds—particularly the degradation caused by pile-up effects due to system dead time and the potential benefits of photon-number-resolving detectors—remains incomplete and has not been systematically established in prior work. In this work, we present the first theoretical derivation of the Cramér–Rao lower bound (CRLB) for dToF systems explicitly accounting for dead time effects, generalize the analysis to SPADs with photon-number-resolving capabilities, and further validate the results through Monte Carlo simulations and maximum likelihood estimation. Our analysis reveals that pile-up not only reduces the information contained within individual ToF but also introduces a previously overlooked statistical coupling between distance and photon flux rate, further degrading ranging precision. The derived CRLB enables the determination of the optimal optical photon flux, laser pulse width (with FWHM of ≈0.56τ), and ToF quantization resolution that yield the best achievable ranging precision, showing that an optimal precision of approximately $0.53\tau /\sqrt{N}$ remains theoretically achievable, where τ is TDC resolution and *N* is the number of laser pulses. The analysis further quantifies the limited performance improvement enabled by increased photon-number resolution, which exhibits rapidly diminishing returns. Overall, these findings establish a unified theoretical framework for understanding the fundamental limits of SPAD-based dToF LiDAR, filling a gap left by earlier studies and providing concrete design guidelines for the selection of optimal operating points.

## 1. Introduction

Light detection and ranging (LiDAR) technology has gained considerable attention in a wide range of applications, including autonomous driving [1,2,3], robotics [4], and remote sensing [5,6]. At its core, LiDAR determines object distances by measuring the time-of-flight (ToF) of laser pulses reflected from targets. Among the various ToF methods, direct ToF (dToF) approaches—by directly measuring the travel time of laser pulses—are particularly attractive due to their structural simplicity and rapid measurement capabilities [7,8].

A key component of recent advances in dToF systems is the single-photon avalanche diode (SPAD). Operating in Geiger mode, SPADs are capable of detecting individual photons with high temporal resolution and low noise, even under photon-starved conditions [9,10]. However, each SPAD pixel undergoes a so-called quenching process after being triggered, during which it recovers from avalanche and prepares for the next detection event [11]. This recovery period, known as the dead time, represents an interval during which the pixel is inactive and incapable of registering new photon events. Moreover, a single SPAD pixel is inherently binary in its response—it only signals the presence or absence of a photon, without quantifying the number of incident photons. To circumvent this limitation, recent efforts have introduced macro-pixel architectures composed of multiple subpixels [2,12,13,14]. In such configurations, a macro-pixel consisting of *s* subpixels can potentially register between 0 and *s* triggered subpixels in a single measurement, thereby endowing the detector with *s*-photon-number resolution (*s*-PNR). This additional degree of photon number information not only extends the system’s dynamic range but also holds promise for improving ranging precision by capturing richer photon statistics.

Another critical component of dToF systems is the time-to-digital converter (TDC), which quantizes the temporal interval between the laser emission and the SPAD detection event [15,16]. A key figure of merit for TDCs is their time resolution, which governs the precision of ToF quantization. However, TDCs are also subject to a form of dead time, imposed by circuit constraints, during which further conversions are temporarily inhibited. Early TDCs were only capable of registering a single event per measurement cycle, which can be considered as an infinite dead time. Advances in circuit design have since allowed for multiple ToF conversions within a single cycle [14,17]. The so-called multi-event TDC increases the available photon timing information and, in principle, can further enhance the system’s ranging accuracy.

In practice, photon detections are inevitably contaminated by noise, including ambient irridiation and circuit noise. To improve the signal-to-noise ratio, modern dToF systems typically emit a sequence of laser pulses and compile a histogram of the corresponding quantized ToF values in which the bin width equals the TDC resolution. Ideally, the bin with the highest count corresponds to the estimated ToF. To further improve estimation accuracy to sub-bin resolution, previous studies have proposed various techniques such as curve-fitting to achieve sub-bin precision by fully exploiting the histogram peak shape [18,19].

A rigorous theoretical analysis of ranging precision is essential for guiding the architectural and algorithmic design of dToF LiDAR systems. While prior work has provided valuable insights into this area [19,20,21], two key aspects remain insufficiently explored. Firstly, the distortion of histogram peak shapes induced by dead time is not fully accounted for, which leads to a deviation between the location of the histogram peak and the ToF corresponding to the true distance. This phenomenon is commonly referred to as the “walk error”. Several calibration methods have been proposed to correct walk errors [22,23,24,25,26], yet the fundamental performance bounds of such correction remain unknown. Secondly, the benefits conferred by macro-pixel architectures with photon-number resolution have not been rigorously characterized from a theoretical perspective.

In this work, the ranging performance bounds of dToF systems is theoretically analyzed based on the fisher information theory by explicitly incorporating the impact of dead time, and the result is generalized to include photon-number-resolving SPADs. Specifically, we derive the probability density functions of detection histograms under various dToF configurations and compute the corresponding Cramér–Rao lower bound (CRLB) on ranging precision. To validate the theoretical results, Monte Carlo (MC) simulations are conducted to generate detection histograms, from which maximum likelihood estimation (MLE) is performed to infer distance. The simulation results exhibit excellent agreement with theory, confirming the robustness and accuracy of our modeling approach.

Our derivations reveal that the presence of dead time leads to additional degradation in the theoretical ranging precision compared to idealized models. Furthermore, the analysis reveals that excessive received pulse intensities can severely deteriorate ranging performance, whereas an optimal received intensity exists that minimizes the CRLB under given system constraints. As a result, an optimal precision of approximately $0.53\tau /\sqrt{N}$ for a 1-PNR detector remains theoretically achievable by appropriately selecting the laser pulse width with FWHM of about 0.56τ and the received photon flux rate, where τ is TDC resolution and *N* is the number of laser pulses. In the case of *s*-PNR SPAD macro-pixels, it is found that the full potential of photon-number resolution can be realized only when the total number of TDC triggers per measurement cycle is also available in addition to the commonly used histogram of total sub-pixel counts. Moreover, increasing photon-number resolution yields only marginal improvements in the system’s theoretical best-case ranging performance.

In conclusion, this work establishes, for the first time, a unified theoretical framework that rigorously accounts for dead-time-induced pile-up and photon-number resolution, thereby clarifying the fundamental performance boundaries of SPAD-based dToF LiDAR. Compared with prior studies that focused on empirical observations or partial models, our results uncover previously overlooked statistical couplings, quantify the limited benefits of photon-number-resolving detectors, and provide concrete guidelines for designing future high-precision, photon-efficient ranging systems.

The remainder of this paper is organized as follows. Section 2 introduces the detection probability model and derives the Fisher information and Cramér–Rao lower bounds for both 1-PNR and *s*-PNR systems. Section 3 applies these theoretical results to performance analysis, including a comparison between 1-PNR and *s*-PNR systems and a representative example illustrating the framework’s practical relevance. Finally, Section 4 concludes the paper.

## 2. Materials and Methods

### 2.1. Detection Model

We begin by modeling SPAD-based dToF LiDAR systems without incorporating the technical details of acquisition electronics or specific measurement techniques. While prior work has derived key results such as the expected histogram counts via single-pulse detection analysis [15,27], here the results are derived from first principles by obtaining the histogram’s full probability distribution, thereby providing the basis for CRLB derivation in the next section. For simplicity, the SPAD dead time is assumed to be equal to the TDC dead time throughout the main text, denoted as T, to avoid the complex histogram behaviors that can arise when the SPAD dead time is shorter than that of the TDC [28], which would reduce the effective photon count and degrade the ranging performance [For the case where the two dead times are unequal, see Section S1 of the Supplementary Information (SI)]. Moreover, since the focus of this work is on analyzing the fundamental limits of ranging accuracy, the model in the main text does not account for non-ideal effects such as afterpulsing, crosstalk, or other circuit-level impairments that may degrade ranging performance in practical implementations [Cases accounting for these non-ideal effects are detailed in Sections S2–S4 of the Supplementary Information (SI)] [29,30].

Let the normalized waveform of the emitted laser pulse be denoted by f(t), such that max{f(t)}=1 and f(t<0)=0. To prevent a ambiguity in ToF estimation introduced by a single laser pulse from generating multiple peaks in the histogram, it is further assumed that the duration of the laser pulse is shorter than the system’s dead time, i.e., f(t>T−1)=0. It should be noted that, upon completion of the analysis, the theoretical results are also applicable to cases with multiple object peaks.

Upon reflection from a target located at distance *z*, the pulse returns to the dToF system and is focused onto the SPAD pixel, generating a signal photon flux of $Rf(t-t_{0})$, where $t_{0}=2z/c$ is the round-trip ToF, *c* is the speed of light. *R* denotes the peak photon flux rate absorbed by the SPAD, which represents the rate of return photons reflected from the target, transmitted through the receiving optics, incident on the SPAD active area, and, after accounting for the SPAD detection efficiency (PDE), capable of triggering an avalanche. The value of *R* can be calculated using the LiDAR equation [27]. For instance, when the target is a uniformly diffuse reflector and the laser spot fully lies within the receiver field of view, *R* can be expressed as

(1) $$R=\eta_{s}\eta_{o}Pr\frac{D^{2}}{4z^{2}}$$

where *P* is the peak power of the emitted laser pulse, *r* is the target reflectivity, *D* is the diameter of the receiving lens, $\eta_{s}$ denotes the PDE, and $\eta_{o}$ represents the overall optical transmittance of the system. The mean photon count of the signal within the time interval of the *i*-th histogram bin is expressed by

(2) $$S_{i}=R\int_{i\tau }^{(i+1)\tau }f\left(t-t_{0}\right)dt,$$

where τ denotes the bin width of the histogram, i.e., the resolution of the TDC. Similarly, the average photon count of noise is given by $b=R_{n}\tau$, where $R_{n}$ denotes the average photon flux of noise absorbed by the SPAD. For coherent light, the photon counts follow a Poisson distribution. Therefore, the probability of no detection event occurring in the *i*-th bin, denoted as $p_{i}$, is given by $p_{i}=e^{-S_{i}-b}$. Correspondingly, the probability of a detection event occurring in the *i*-th bin is given by $q_{i}=1-p_{i}$.

For a SPAD detector without PNR capability, i.e., a 1-PNR SPAD, let the system perform *N* independent pulse measurements, accumulating the histogram h=[h(0),h(1),…]. If the counts in the previous T=T/τ bins are known as $h_{i-1},h_{i-2},\ldots ,h_{i-T}$, then due to the dead time constraint, the count h(i) in the *i*-th bin follows a conditional binomial distribution:

(3) $$P\left\{h\left(i\right)=h_{i}|h\left(i-1\right)=h_{i-1},h\left(i-2\right)=h_{i-2},\ldots ,h\left(i-T\right)=h_{i-T}\right\}∼B\left(N_{i}^{\prime },q_{i}\right),$$

where $N_{i}^{\prime }$ is defined as

(4) $$N_{i}^{\prime }=N-\sum_{j=1}^{T}h_{i-j}.$$

This formulation reflects the physical constraint imposed by the detector dead time: only those measurements that did not trigger in the previous *T* bins remain available to contribute at bin *i*. Hence, $N_{i}^{\prime }$ denotes the number of ’active’ trials at bin *i*, while $q_{i}$ represents the probability that an active trial produces a detection. As a result, h(i) follows a conditional binomial distribution $B(N_{i}^{\prime },q_{i})$. For simplicity, this conditional probability is denoted as $H_{i}\left(h_{i}|h_{i-1},h_{i-2},\ldots ,h_{i-T}\right)$.

With the law of total expectation, the expectation of $P\{h\left(i\right)=h_{i}\}$ is given by

(5) $$E\left[h\left(i\right)\right]=q_{i}E\left[N_{i}^{\prime }\right]=q_{i}\left(N-\sum_{j=1}^{T}E\left[h\left(i-j\right)\right]\right).$$

Defining $E\left[h\left(i\right)\right]=NQ_{i}=NF_{i}q_{i}$, and $Q_{i}=F_{i}q_{i}$ represents the expected count in the *i*-th bin for a single measurement. It can be seen that this expectation is proportional to the probability of generating a detection event, $q_{i}$, multiplied by the correction factor $F_{i}$, which accounts for the reduced number of measurable pulses due to dead time, reflecting the so-called “pile-up” effect. Compared with Equation (5), the expression for $F_{i}$ can be derived as

(6) $$F_{i}=1-\sum_{j=1}^{T}Q_{i-j},$$

which indicates that the $F_{i}$ equals the probability that the *i*-th bin is not within the dead time caused by triggers in the preceding *T* bins. It can be further expressed as a recursive relation

(7) $$F_{i}=F_{i-1}-Q_{i-1}+Q_{i-T-1}=F_{i-1}p_{i-1}+Q_{i-T-1}.$$

This recursion can be interpreted as follows: the probability that the *i*-th bin is available for detection equals the probability that the previous bin was available but did not generate a detection event, plus the probability that a detection occurred in the (i−T−1)-th bin, whose dead time ends exactly at the (i−1)-th bin. If the system uses a single-TDC architecture, where the TDC triggers only once per laser pulse measurement cycle, then the recursive relation can be written as

(8) $$F_{i}=F_{i-1}-Q_{i-1}=F_{i-1}p_{i-1}.$$

Compared with Equation (7), it can be seen that, because the TDC can register at most one count per laser pulse measurement cycle here, the detection probability of the *i*-th bin equals only the probability that the previous bin was available for detection but did not generate a detection event.

For the boundary conditions of the above recursion, before each measurement cycle starts, the SPAD is in a steady state with only background noise present; therefore, the probability of the SPAD being triggered at each “negative bin” with i<0 should be equal. Denoting $q_{b}=1-e^{-b}$ as the probability of a trigger in a single bin due to background noise, without accounting for the dead-time effect, the actual probability *Q* of these bins being triggered within a single pulse measurement is reduced ($Q<q_{b}$) because of the dead time that occurred in the preceding *T* bins. This probability must also be multiplied by the reduction factor $F_{b}$. There are three possible conditions for a single “steady bin”: (1) Within the dead time caused by the preceding detection event occured in preceding *T* bins, with the probability of $q_{b}F_{b}T$; (2) Not affected by dead time, and generating a detection event, with the probability of $q_{b}F_{b}$; (3) Not affected by dead time, and not generating a detection event, with the probability of $F_{b}\left(1-q_{b}\right)$. With normalized condition $q_{b}F_{b}+\left(1-q_{b}\right)F_{b}+q_{b}F_{b}T=1$, it results in $F_{b}=1/\left(1+q_{b}T\right)$. With $E\left[h\left(i<0\right)\right]=NQ_{i}$, the above result implies that $Q_{b}=q_{b}F_{b}=q_{b}/\left(1+q_{b}T\right)$, which aligns with the results previously reported in the literature [15] [see Appendix A for the details].

For *s*-PNR SPADs, which consist of *s* identical sub-pixels, it is assumed that the incident light is uniformly distributed among all sub-pixels, yielding an instantaneous photon flux rate per sub-pixel of $R^{\prime }=R/s$. During the time interval corresponding to the *i*-th bin, the probability that a sub-pixel does not and does generate a detection event is given by $v_{i}=e^{-(S_{i}+b)/s}$ and $u_{i}=1-v_{i}$, respectively. To simplify the analysis and enable analytical derivation, we further assume synchronous triggering across sub-pixels [see Section S5 of Supplementary Information (SI)]. For each laser pulse measurement, the detector records the number of sub-pixels triggered and accumulates this count into the histogram, denoting as k(i). Similar to Equation (3), due to the constraint imposed by dead time, calculating the total count k(i) in the *i*-th bin requires knowledge of the number of TDC triggers that occurred within the preceding *T* bins. These TDC trigger counts are equivalent to the counts obtained by treating the *s*-PNR SPAD as a conventional 1-PNR SPAD, so they are also denoted as $h_{i}$. The conditional probability of observing a total of $k_{i}$ triggered subpixels in the *i*-th bin, given $h\left(i\right)=h_{i}$ TDC triggers, is expressed as:

(9) P{k(i)=ki|h(i)=hi,h(i−1)=hi−1,…,h(i−T)=hi−T}=Ni′hiJs(hi,ki)uikivisNi′−ki,

where Ni′hi is the binomial coefficient, $N_{i}^{\prime }$ is defined as in Equation (4), and $J_{s}\left(h_{i},k_{i}\right)$ denotes the number of ways to allocate $k_{i}$ triggered subpixels across $h_{i}$ TDC events, under the constraint that no more than *s* subpixels can be triggered per event. $J_{s}$ further satisfies

(10) ∑hi=0Ni′Ni′hiJs(hi,ki)=sNi′ki.

Therefore, marginally, the total photon count k(i) follows the binomial distribution:

(11) $$P\left(k\left(i\right)|N_{i}^{\prime }\right)∼B\left(sN_{i}^{\prime },u_{i}\right).$$

Using the law of total expectation and referring to Equation (5), the mean of k(i) across *N* measurements can be written as

(12) $$E\left[k\left(i\right)\right]=sNQ_{i}=sNF_{i}u_{i},$$

where $F_{i}$ remains defined as in Equation (6).

**Figure 1:**
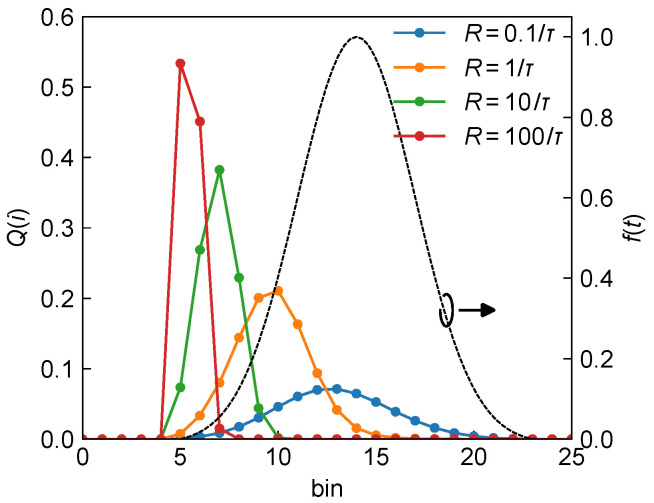
Theoretical histogram expectation and pile-up effect. Black dashed line (right axis): the theoretical laser pulse waveform $f(t-t_{0})$ corresponding to a ToF of $t_{0}=5$. Solid lines (left axis): expected single-pulse histograms $Q_{i}$ under different peak received photon flux rates with R=0.1/τ (blue), 1/τ (orange), 10/τ (green), and 100/τ (red).

Figure 1 illustrates the expected histograms $Q_{i}$ corresponding to a Gaussian laser pulse reflected from targets located at the same distance $t_{0}$, but with varying reflectivity levels, resulting in different photon flux rates *R*, in a dToF system using a 1-PNR SPAD. As *R* increases, the temporal bias of the detection histogram progressively moves to the left and becomes narrower, indicating a more severe pile-up effect caused by the detector’s dead time. If the target distance is estimated directly from the peak position in the histogram, this bias leads to a walk error, where targets at the same physical distance but with different reflectivities are erroneously estimated to be at different distances.

It should be noted that both here and in the following, *R* is expressed in units of the inverse TDC time resolution, 1/τ. According to the LiDAR equation (Equation (1)), *R* depends on multiple system parameters, and its value can span several orders of magnitude across different dToF architectures. For example, in the single-point scanning dToF system discussed in [31], *R* typically varies from about $10^{-1}$∼$10^{5}/\tau$, depending on laser pulse energy and target distance. As such, *R* acts as a key intermediate parameter in system design, enabling the determination of other optical and electrical design choices. While our analysis here emphasizes the theoretical framework of dToF systems in a system-agnostic fashion, we note that in practical automotive scenarios, typical reflectivities specified in standards (e.g., 10%, 50%, and 90% [32,33,34]) or those of other common objects (e.g., road signs or asphalt [35,36]) can be directly mapped to corresponding photon flux levels according LiDAR equation (Equation (1)) once the system parameters (such as laser power and receiver aperture) are fixed. This establishes a direct link between the theoretical framework presented here and system-level evaluations.

### 2.2. Cramér–Rao Lower Bound

Based on the system’s probabilistic model derived earlier, the theoretical ranging performance, or specifically, the Cramér–Rao lower bound (CRLB) for the variance of the ToF estimate $\mathrm{Var}\{\hat{t_{0}}\}$, can be calculated. To evaluate the fundamental performance limit of detection, it is essential to minimize the number of unknown system parameters to be estimated other than $t_{0}$. As discussed in the previous section, the system output (i.e., the histogram) is primarily influenced by two categories of parameters. The first category consists of parameters that are intrinsic to the detection system and independent of the measurement environment, including the dead time *T*, TDC resolution τ, and the laser pulse waveform f(t), which all can be accurately characterized through system design and calibration. The second category comprises environment-dependent parameters, such as the background noise level $R_{n}$, the target distance (i.e., ToF $t_{0}$), and the peak received photon rate *R*. Among these parameters, the background noise rate $R_{n}$, which includes ambient light and dark noise, can be reliably estimated via long-duration passive measurements by the dToF system (e.g., acquiring histograms without emitting laser pulses), and thus is assumed to be known. Given the lack of prior knowledge about the target object, the set of unknown parameters to be estimated is defined as $\theta =(t_{0},R)$.

Assuming that the histogram contains only a single target-induced peak located at $t_{0}$, the probability of observing a specific histogram realization with $h\left(0\right)=h_{0},h\left(1\right)=h_{1},\ldots ,h\left(l\right)=h_{l}$ is given by

(13) $$\begin{array}{ccc} & & P(h_{l};\theta )\\ & = & P\{h\left(0\right)=h_{0},h\left(1\right)=h_{1},\ldots ,h\left(l\right)=h_{l}\}\\ & = & \sum_{m}P\left\{h\left(0\right)=h_{0},h\left(1\right)=h_{1},\ldots ,h\left(l\right)=h_{l}|h\left(-1\right)=m_{1},h\left(-2\right)=m_{2},\ldots ,h\left(-T\right)=m_{T}\right\}\times \\ & & P\{h\left(-1\right)=m_{1},h\left(-2\right)=m_{2},\ldots ,h\left(-T\right)=m_{T},h\left(-T-1\right)=m_{T+1}\}\\ & = & E_{m}\left[H_{0}H_{1}\ldots H_{l}\right]\\ & \overset{\mathrm{def}}{=} & {\tilde{H}}_{l}, \end{array}$$

where $h_{l}=\left(h_{0},h_{1},\ldots ,h_{l}\right)$ and $E_{m}\left[\cdot \right]$ is the expection over all possible m.

According to the Cramér–Rao theorem [37], the covariance matrix $C_{\hat{\theta }}$ of any unbiased estimator $\hat{\theta }$ satisfies

(14) $$C_{\hat{\theta }}\geq I\left(\theta \right),$$

where I(θ), the Fisher information matrix for θ, is expressed as

(15) $$I_{ij}\left(\theta \right)=\sum_{h_{l}}P\left(h_{l};\theta \right)\left(\frac{\partial \ln P(h_{l};\theta )}{\partial \theta_{i}}\right)\left(\frac{\partial \ln P(h_{l};\theta )}{\partial \theta_{j}}\right).$$

As the focus is on the estimation accuracy of $t_{0}$, it follows that

(16) $$\mathrm{Var}\left\{\hat{t_{0}}\right\}={\left[I{\left(t_{0},R\right)}^{-1}\right]}_{11}.$$

Thus, it is necessary to compute the elements of the Fisher information matrix $I(t_{0},R)$.

The simplification for an arbitrary element in the matrix (Equation (15)) can be expressed as

(17) $$\begin{array}{ccc} & & \sum_{h_{l}}P\left(h_{l};\theta \right)\left(\frac{\partial \ln P(h_{l};\theta )}{\partial \theta_{i}}\right)\left(\frac{\partial \ln P(h_{l};\theta )}{\partial \theta_{j}}\right)\\ & = & \sum_{h_{l}}\frac{1}{{\tilde{H}}_{l}}\left(\frac{\partial {\tilde{H}}_{l}}{\partial \theta_{i}}\right)\left(\frac{\partial {\tilde{H}}_{l}}{\partial \theta_{j}}\right)\\ & = & \sum_{h_{l}}\frac{1}{{\tilde{H}}_{l}}\left({\tilde{H}}_{l-1}\frac{\partial H_{l}}{\partial \theta_{i}}+\frac{\partial {\tilde{H}}_{l-1}}{\partial \theta_{i}}H_{l}\right)\left({\tilde{H}}_{l-1}\frac{\partial H_{l}}{\partial \theta_{j}}+\frac{\partial {\tilde{H}}_{l-1}}{\partial \theta_{j}}H_{l}\right)\\ & = & \sum_{h_{l}}\left({\tilde{H}}_{l-1}\frac{1}{H_{l}}\frac{\partial H_{l}}{\partial \theta_{i}}\frac{\partial H_{l}}{\partial \theta_{j}}+\frac{1}{{\tilde{H}}_{l-1}}H_{l}\frac{\partial {\tilde{H}}_{l-1}}{\partial \theta_{i}}\frac{\partial {\tilde{H}}_{l-1}}{\partial \theta_{j}}+\left(\frac{\partial {\tilde{H}}_{l-1}}{\partial \theta_{i}}\frac{\partial H_{l}}{\partial \theta_{j}}+\frac{\partial H_{l}}{\partial \theta_{i}}\frac{\partial {\tilde{H}}_{l-1}}{\partial \theta_{j}}\right)\right). \end{array}$$

It can be seen that the expanded Fisher information consists of three terms. The first term represents the information obtained from the current bin (the *l*-th bin). The second term accounts for the information contributed by previous bins (those with indices less than *l*). The third term captures the possible coupling effects between the current bin and the previous bins.

For the first term, the derivation term can be simplified according to Equation (3) as

(18) $$\frac{\partial H_{l}}{\partial \theta_{i}}=H_{l}\frac{h_{l}-N_{l}^{\prime }q_{l}}{p_{l}q_{l}}\frac{\partial q_{l}}{\partial \theta_{i}}.$$

So the first term results in [see Appendix B]

(19) $$\sum_{h_{l}}{\tilde{H}}_{l-1}\frac{1}{H_{l}}\frac{\partial H_{l}}{\partial \theta_{i}}\frac{\partial H_{l}}{\partial \theta_{j}}=N\frac{F_{l}}{p_{l}q_{l}}\frac{\partial q_{l}}{\partial \theta_{i}}\frac{\partial q_{l}}{\partial \theta_{j}}.$$

The second term simplifies to the sum of the Fisher information from bin 0 to bin l−1:

(20) $$\begin{array}{ccc} & & \sum_{h_{l}}\frac{1}{{\tilde{H}}_{l-1}}H_{l}\frac{\partial {\tilde{H}}_{l-1}}{\partial \theta_{i}}\frac{\partial {\tilde{H}}_{l-1}}{\partial \theta_{j}}\\ & = & \sum_{h_{l-1}}\frac{1}{{\tilde{H}}_{l-1}}\frac{\partial {\tilde{H}}_{l-1}}{\partial \theta_{i}}\frac{\partial {\tilde{H}}_{l-1}}{\partial \theta_{j}}\sum_{h_{l}}H_{l}\\ & = & \sum_{h_{l-1}}\frac{1}{{\tilde{H}}_{l-1}}\frac{\partial {\tilde{H}}_{l-1}}{\partial \theta_{i}}\frac{\partial {\tilde{H}}_{l-1}}{\partial \theta_{j}}\\ & = & \sum_{h_{l-1}}P\left(h_{l-1};\theta \right)\left(\frac{\partial \ln P(h_{l-1};\theta )}{\partial \theta_{i}}\right)\left(\frac{\partial \ln P(h_{l-1};\theta )}{\partial \theta_{j}}\right). \end{array}$$

And the third term is simply zero [see Appendix B].

Combining the above results, the Fisher information simplifies to

(21) $$I_{ij}\left(\theta \right)=N\sum_{k=0}^{l}\frac{F_{k}}{p_{k}q_{k}}\frac{\partial q_{k}}{\partial \theta_{i}}\frac{\partial q_{k}}{\partial \theta_{j}}.$$

Let $l_{1}=\left\lfloor t_{0}/\tau \right\rfloor$ denote the first bin containing the returning laser pulse and $l_{2}=l_{1}+T$. Since $\partial q_{k}/\partial \theta_{i}\neq 0$ only when $l_{1}\leq k\leq l_{2}$, and for the Fisher information is proportional to the number of measurements *N*, the Fisher information matrix can be expressed as

(22) $$I_{ij}\left(\theta \right)=NI_{ij}\left(\theta \right)=N\sum_{k=l_{1}}^{l_{2}}\frac{F_{k}}{p_{k}q_{k}}\frac{\partial q_{k}}{\partial \theta_{i}}\frac{\partial q_{k}}{\partial \theta_{j}},$$

where I denotes the Fisher information corresponding to a single pulse measurement. It can be noted that each term in the sum exactly corresponds to the Fisher information contributed by the *k*-th histogram bin when the counts follow a binomial distribution $B(NF_{k}(\theta ),q_{k}\left(\theta )\right)$ with respect to estimating the parameter θ.

According to Equation (16), the lower bound of variance of the estimator for the ToF $t_{0}$ is given by the (1,1)-th element of the inverse Fisher information matrix:

(23) $$\mathrm{Var}\left\{\hat{t_{0}}\right\}={\left[I{\left(t_{0},R\right)}^{-1}\right]}_{11}=\frac{1}{N\left[I_{11}-\frac{I_{12}^{2}}{I_{22}}\right]}$$

It is clear that the variance of the estimator for $t_{0}$ decreases inversely with *N*. Define the correlation coefficient between the two parameters as

(24) $$\rho^{2}=\frac{I_{12}^{2}}{I_{11}I_{22}},$$

then, the above expression can be rewritten as

(25) $$\mathrm{Var}\left\{\hat{t_{0}}\right\}=\frac{1}{NI_{11}\left(1-\rho^{2}\right)}.$$

From Equations (22) and (25), the presence of dead time degrades the variance of $t_{0}$ estimation in two ways. On the one hand, each histogram bin’s individual Fisher information contribution is weighted by the factor $F_{i}$. Since $F_{i}\leq 1$ and is monotonically decreasing, bins corresponding to later times contribute less information. On the other hand, the dead time-induced pile-up effect introduces correlation ρ between the estimates of $t_{0}$ and *R*, which further degrades the variance of $t_{0}$ estimation as ρ increases. When ρ=0, the estimations of the two parameters are statistically independent, yielding $\mathrm{Var}\left\{\hat{t_{0}}\right\}=1/\left(NI_{11}\right)$, meaning the variance of $t_{0}$ is unaffected by whether *R* is known or not. As ρ increases, the correlation between the two parameter estimates increases, which leads to degraded estimation performance for $t_{0}$. When ρ is approaching 1, the estimates become fully correlated and $\mathrm{Var}\{\hat{t_{0}}\}$ diverges implying that effective estimation of $t_{0}$ is impossible.

Here, our results are compared with those reported in Refs. [19,20]. In these references, the effects of dead time were not considered, resulting in complete decoupling between the estimation of $t_{0}$ and *R*. Moreover, the return intensity of the laser waveform was assumed to be linearly related to the histogram peak height, without accounting for the SPAD’s nonlinear response to high photon flux. Under these approximations, the ToF estimation reduces to estimating the center of a Gaussian peak in the time-varying return signal. Consequently, the estimation depends on the Gaussian peak width and the TDC bin width, with performance improving as the signal strength increases, eventually being limited by the TDC bin width. The CRLB obtained using this approach approximates the term $1/\sqrt{I_{11}}$ derived in the present work with all $F_{i}=1$. However, because the current study accounts for the SPAD’s nonlinear response to high photon flux, the CRLB reported in the references is more optimistic when *R* is large.

Similarly, for the *s*-PNR SPAD detector, we denote Equation (9) as $K_{i}\left(k_{i}|h_{i},h_{i-1},\ldots ,h_{i-T}\right)$ and the probability of observing a specific histogram with photon number counts $k_{l}=\left[k\left(1\right)=k_{1},k\left(2\right)=k_{2},\ldots \right]$ and corresponding TDC trigger counts $h_{l}=\left[h\left(1\right)=h_{1},h\left(2\right)=h_{2},\ldots \right]$ is given by

(26) $$P\left(k_{l},h_{l};\theta \right)=E_{m}\left[K_{1}K_{2}\ldots K_{l}\right]\overset{\mathrm{def}}{=}{\tilde{K}}_{l}.$$

And the fisher information matrix is defined as

(27) $$I_{ij}\left(\theta \right)=\sum_{k_{l},h_{l}}P\left(k_{l},h_{l};\theta \right)\left(\frac{\partial \ln P(k_{l},h_{l};\theta )}{\partial \theta_{i}}\right)\left(\frac{\partial \ln P(k_{l},h_{l};\theta )}{\partial \theta_{j}}\right).$$

Similarly to Equation (17), each element of the Fisher information matrix can be decomposed into three terms as follows:

(28) $$\begin{array}{ccc} & & \sum_{k_{l},h_{l}}P\left(k_{l},h_{l};\theta \right)\left(\frac{\partial \ln P(k_{l},h_{l};\theta )}{\partial t_{0}}\right)\left(\frac{\partial \ln P(k_{l},h_{l};\theta )}{\partial R}\right)\\ & = & \sum_{k_{l},h_{l}}\left({\tilde{K}}_{l-1}\frac{1}{K_{l}}\frac{\partial K_{l}}{\partial \theta_{i}}\frac{\partial K_{l}}{\partial \theta_{j}}+\frac{1}{{\tilde{K}}_{l-1}}K_{l}\frac{\partial {\tilde{K}}_{l-1}}{\partial \theta_{i}}\frac{\partial {\tilde{K}}_{l-1}}{\partial \theta_{j}}+\left(\frac{\partial {\tilde{K}}_{l-1}}{\partial \theta_{i}}\frac{\partial K_{l}}{\partial \theta_{j}}+\frac{\partial K_{l}}{\partial \theta_{i}}\frac{\partial {\tilde{K}}_{l-1}}{\partial \theta_{j}}\right)\right) \end{array}$$

The derivative term in the first term is calculated as

(29) $$\frac{\partial K_{l}}{\partial \theta_{i}}=K_{l}\frac{k_{l}-sN_{l}^{\prime }u_{l}}{v_{l}u_{l}}\frac{\partial u_{l}}{\partial \theta_{i}}.$$

So similarly with Equation (19), the first term simplifies to

$$\sum_{k_{l},h_{l}}{\tilde{K}}_{l-1}\frac{1}{K_{l}}\frac{\partial K_{l}}{\partial \theta_{i}}\frac{\partial K_{l}}{\partial \theta_{j}}=sN\frac{F_{l}}{v_{l}u_{l}}\frac{\partial u_{l}}{\partial \theta_{i}}\frac{\partial u_{l}}{\partial \theta_{j}}.$$

The second and third terms can be further simplified, similarly to what was done in the 1-PNR case. Therefore, the final Fisher information matrix is simplified as

(30) $$I_{ij}\left(\theta \right)=sN\sum_{k=l_{1}}^{l_{2}}\frac{F_{k}}{v_{k}u_{k}}\frac{\partial u_{k}}{\partial \theta_{i}}\frac{\partial u_{k}}{\partial \theta_{j}}.$$

Similar to Equation (22), each term in Equation (30) exactly corresponds to the Fisher information for estimating θ when the count in the *k*-th bin of the histogram follows a binomial distribution $B(sNF_{k}\left(\theta \right),u_{k}\left(\theta \right))$. Clearly, when s=1, Equation (30) reduces to the same result as the ranging performance for detectors without photon-number resolution, as given in Equation (22).

In the above derivation, both the total subpixel-triggered histogram k and the TDC trigger counts h are assumed to be known. However, many current dToF systems based on multi-subpixel SPAD architectures only record k without storing h. The absence of h clearly leads to a loss of Fisher information, which in turn degrades the estimation performance for $t_{0}$. To distinguish between these two system architectures, we define systems that record both k and h as Type I, and those that record only k as Type II. For Type II systems, the Fisher information becomes

(31) $$I_{ij}\left(\theta \right)=\sum_{k_{l}}\left[\sum_{h_{l}}P\left(k_{l},h_{l};\theta \right)\left(\sum_{h_{l}}\frac{\partial \ln P(k_{l},h_{l};\theta )}{\partial \theta_{i}}\right)\left(\sum_{h_{l}}\frac{\partial \ln P(k_{l},h_{l};\theta )}{\partial \theta_{j}}\right)\right].$$

Since h becomes latent variables in this case, the above expression is difficult to simplify into a clear closed form. Here, an asymptotic expression for large *N* will be derived to evaluate the system performance in the limit [see Section S6 of Supplementary Information (SI)].

To derive the asymptotic expression, the random vector k(i) is approximated asymptotically by a multivariate normal distribution. For the expectation, $E\left[k\left(i\right)\right]=sNF_{i}u_{i}$ from Equation (12). Due to the absence of trigger count information h, whether the *i*-th bin is within the dead time follows a Bernoulli distribution with probability $F_{i}$. According to the total variance equation, the variance of k(i) is

(32) $$\begin{array}{ccc} \mathrm{Var}\{k(i)\} & = & \mathrm{Var}\left\{B\left(sNF_{i},u_{i}\right)\right\}+{\left(su_{i}\right)}^{2}\mathrm{Var}\left\{B\left(N,F_{i}\right)\right\}\\ & = & sNF_{i}u_{i}\left(1-u_{i}\right)+s^{2}u_{i}^{2}NF_{i}\left(1-F_{i}\right)\\ & = & sNC_{ii}, \end{array}$$

where $C_{ii}=F_{i}u_{i}\left(1-u_{i}\right)+su_{i}^{2}F_{i}\left(1-F_{i}\right)$. For the covariance between k(i) and k(i), let Y(i) denote the random variables of SPADs triggered in a single detection event at the *i*-th bin. Due to the presence of dead time, when i≠j and |i−j|≤T, it holds Y(i)Y(j)=0, hence

(33) $$\begin{array}{ccc} \mathrm{Cov}(k(i),k(j)) & = & N\mathrm{Cov}(Y(i),Y(j))=N(E[Y(i)Y(j)]-E[Y(i)]E[Y(j)])\\ & = & -NE\left[Y\left(i\right)\right]E\left[Y\left(j\right)\right]=-Ns^{2}F_{i}F_{j}u_{i}u_{j}=sNC_{ij} \end{array}$$

where $C_{ij}=-sF_{i}F_{j}u_{i}u_{j}$. Compared with Type I system, the presence of $F_{i}\left(1-F_{i}\right)$ term in variance for counts in each bin and covariance between bins in Type II systems prevents direct inference of whether each bin was affected by the dead time during individual measurements based solely on the histogram data k. This inter-bin correlation reduces the overall obtainable Fisher information.

So in the large-*N* limit, the joint distribution of $k=(k_{0},k_{1},\ldots )$ converges to a multivariate normal distribution N(sNQ,sNC) where $Q=[Q_{0},Q_{1},\ldots ]$. The Fisher information for the Type II system is ultimately expressed as [37,38]

(34) $$I_{ij}\left(\theta \right)\approx sN\frac{\partial Q(\theta )}{\partial \theta_{i}}C{\left(\theta \right)}^{-1}\frac{\partial Q(\theta )}{\partial \theta_{j}}+O\left(1\right).$$

where the explicit expression of $C^{-1}$ is shown in Appendix C. Since the first term is proportional to sN, it dominates when *N* is large.

**Figure 2:**
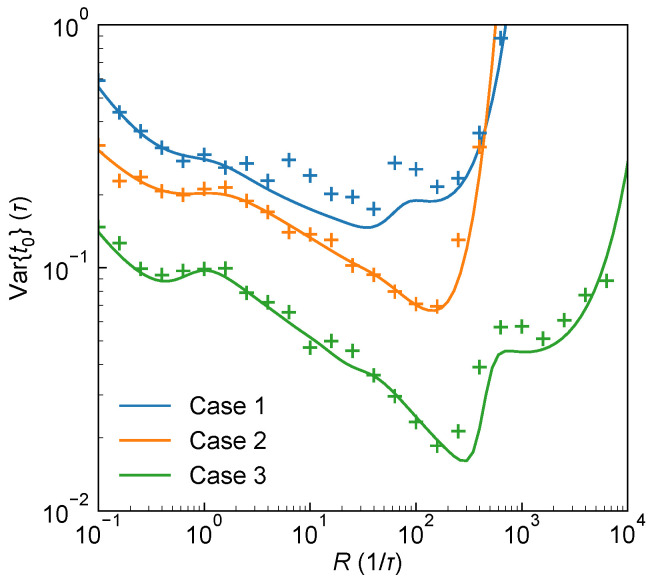
Theoretical ranging precision $\mathrm{Var}\{\hat{t_{0}}\}$ as a function of the peak received photon flux rate *R* under three system architectures. Solid lines represent theoretical calculations, and crosses denote the variances of 100 sets of $t_{0}$ estimates obtained via MLE from histograms generated by MC simulations at different values of *R*. f(t) is assumed as a Gaussian-shaped laser pulse with full width at half maximum (FWHM) w=4τ. Case 1 (blue): 1-PNR SPAD with N=100, $t_{0}=10\tau$ and $R_{n}=0.02/\tau$; Case 2 (orange): 4-PNR SPAD with Type I system, N=100, $t_{0}=10.2\tau$ and $R_{n}=0$; Case 3 (green): 4-PNR SPAD with Type II system, N=1000, $t_{0}=10.5\tau$ and $R_{n}=0.01/\tau$.

In conclution, the above derivations show that the CRLB for estimating $t_{0}$, $\mathrm{Var}\{\hat{t_{0}}\}$, depends on both $t_{0}$ and *R* under all three system architectures (1-PNR SPAD and Type I/II *s*-PNR SPAD systems). To validate the theoretical results, MC simulations were conducted to generate histograms for different system architectures, followed by parameter estimation using the MLE [see Appendix D]. In practical measurements, although the surface reflectivity of the target cannot be controlled, the system can adjust *R* by varying the laser output power, thereby tuning the estimation performance of $t_{0}$. Figure 2 presents typical CRLB behaviors for estimating $t_{0}$ under varying *R* for three detection architectures. It can be seen from the figure that the results show excellent agreement between MC simulations combined with MLE and the theoretical CRLB predictions, confirming the validity of the theoretical analysis.

It should be noted that the CRLB presented here not only provides the theoretical performance bounds for the system’s ranging accuracy, but also serves as a lower bound for the performance of any unbiased dead-time compensation algorithm, meaning that the ranging precision achieved by any unbiased post-processing-based dead-time correction cannot surpass this theoretical limit [see Section S7 of Supplementary Information (SI)].

### 2.3. Section Summary

In this section, a comprehensive probabilistic framework is established for both 1-PNR and *s*-PNR SPAD detectors. For 1-PNR detectors, the detection probability model is developed, describing the conditional probability density function of the histogram counts, h, denoted as *H*. Based on this model, the expected values of the histogram bins, $Q_{i}$, and the corresponding reduction factors, $F_{i}$, are calculated, and is subsequently extended to *s*-PNR detectors. The full probability distribution of the histogram, P{h}, is derived from *H* and is incorporated into the Fisher information $I_{ij}\left(\theta \right)$ calculation, yielding an explicit expression. This derivation is similarly extended to *s*-PNR detectors, considering both Type I and Type II architectures. For Type I, where the number of TDC triggers is known and no latent variables are present, the derivation parallels that of the 1-PNR case. In contrast, Type II lacks TDC trigger information, introducing latent variables; therefore, the system is assumed to follow a multivariate normal distribution under a large-*N* approximation, enabling the Fisher information derivation. Finally, comparison with MC simulations combined with MLE demonstrates excellent agreement, validating the accuracy of the analytical derivations.

## 3. Results

Up to now, the CRLB for all three SPAD-based dToF LiDAR systems are derived. The following section utilizes the analytic results to further analyze the ranging performance of different system architectures and investigate how the systems can achieve their fundamental ranging limits. Since the CRLB scales inversely with the number of detection events *N*, to facilitate intuitive comparison of ranging performance at different *N*, we define $\delta t_{0}=\sqrt{N\times \mathrm{Var}\{\hat{t_{0}}\}}=1/\sqrt{I_{11}\left(1-\rho^{2}\right)}$, representing the lower bound of the standard deviation of the ToF measurement per single pulse measurement at given $t_{0}$ and *R*.

### 3.1. Ranging Performance for 1-PNR SPAD

#### 3.1.1. Performance Evaluation at a Fixed Range

**Figure 3:**
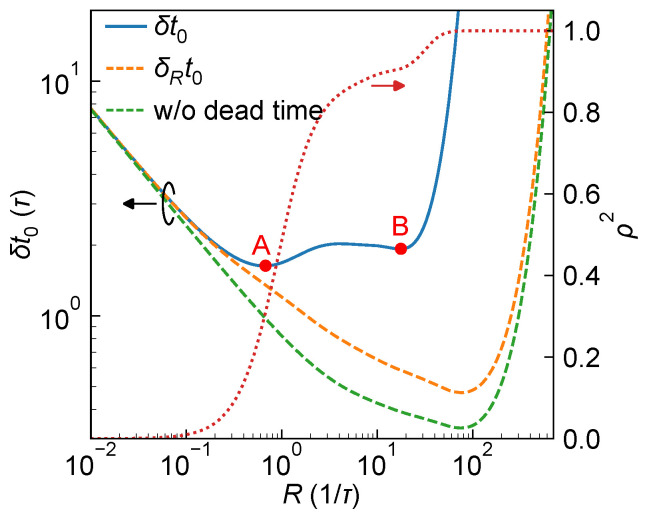
System ranging performance for a Gaussian-shaped laser pulse with FWHM w=3τ and ToF with $t_{0}=10\tau$ as a function of *R*. Variation of system ranging precision $\delta t_{0}$ (blue solid, left axis), ranging precision with known *R*, $\delta_{R}t_{0}$ (orange dashed, left axis), ranging precision without accounting for dead time (green dashed, left axis), and correlation coefficient $\rho^{2}$ (red dotted line, right axis) are shown as functions of *R* for comparison. Two local minima are marked as A and B.

Figure 3 illustrates the dependence of the system ranging precision $\delta t_{0}$ on the peak received photon flux rate *R*, under the condition where the Gaussian-shaped laser pulse has a FWHM of 3τ and the true photon arrival time $t_{0}=10\tau$ [see Section S8 of Supplementary Information (SI)]. For comparison, the figure also plots the lower bound $\delta_{R}t_{0}=1/\sqrt{I_{11}}$, which represents the standard deviation limit for estimating $t_{0}$ assuming a known *R*, as well as the performance neglected the effect of detector dead time (i.e., let $F_{i}=1$ for all bins), which can be regarded as approximately equal to the CRLB lower bound reported in Refs. [19,20]. It can be observed that, when the effect of dead time is neglected, the ranging performance curve lies at the bottom (green dashed line), achieving a minimum value of $\delta t_{0}\approx 0.33\tau$. This value is approximately five times better than the best performance when dead time is considered in this particular case ($\delta t_{0}\approx 1.63\tau$), indicating an overly optimistic estimation of the system’s ranging capability. This discrepancy also highlights the adverse impact of walk error on achievable ToF accuracy.

Specifically, in the regime of small *R*, the ranging precision predicted by all three conditions improves with increasing *R*, which is reasonable: at low photon flux rate, the pixel is rarely triggered, resulting in very limited information content, and a modest increase in *R* significantly enhances the likelihood of photon detection, thereby improving the Fisher information for estimating $t_{0}$. Moreover, due to the negligible effect of temporal pile-up with small *R*, the correction factor $F_{i}\approx 1$ and ρ≈0, leading to nearly identical ranging precision curves predicted by all three conditions.

In contrast, $\delta_{R}t_{0}$ diverges as *R* becomes very large, indicating that even with known *R*, accurate estimation of $t_{0}$ becomes fundamentally infeasible. Intuitively, when *R* is extremely high, nearly all detection events trigger in the first histogram bin where the laser pulse falls, making the peak effectively only one bin wide. This extreme pile-up effect limits the amount of available temporal information, preventing sub-bin estimation of $t_{0}$.

Notably, although $\delta_{R}t_{0}$ may still appear to decrease when R>0.5/τ, $\delta t_{0}$ begins to plateau and eventually diverges beyond R≈30/τ. This behavior is further explained by the evolution of $\rho^{2}$, which begins to increase rapidly beyond R≈0.1/τ and asymptotically approaches unity. The increase in $\rho^{2}$ indicates the emergence and strengthening of pile-up effects, leading to a growing statistical correlation between $t_{0}$ and *R*, which in turn degrades the precision of $t_{0}$ estimation and causes $\delta t_{0}$ to diverge.

In the intermediate region, approximately 0.1/τ≲R≲40/τ, the variation of $\delta t_{0}$ arises from a delicate interplay between $\delta_{R}t_{0}$ and the correlation factor $\rho^{2}$, resulting in a non-monotonic and complex dependence. As shown in the figure, $\delta t_{0}$ exhibits two local minima (marked as points A and B), with the minimum (point A) occurring near R≈0.64/τ and reaching a value of $\delta t_{0}\approx 1.63\tau$. This suggests that, to approach the theoretical limit of ranging performance under this specific condition, the laser output power should be carefully tuned such that the received photon flux rate *R* lies near this optimal value.

**Figure 4:**
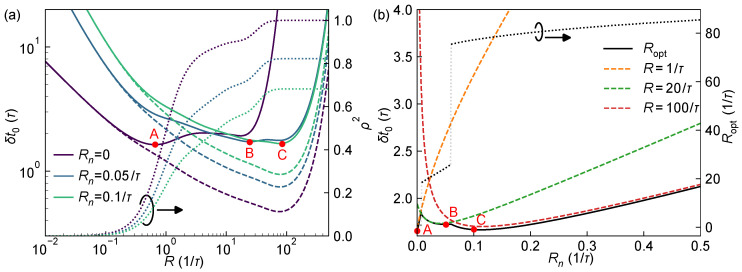
System ranging performance as a function of background noise intensity $R_{n}$ with w=3τ and $t_{0}=10\tau$. (**a**) Variation of system ranging precision $\delta t_{0}$ (solid line, left axis), ranging precision with known *R*, $\delta_{R}t_{0}$ (dashed line, left axis), and correlation coefficient $\rho^{2}$ (dotted line, right axis) as functions of *R*, under different $R_{n}$ levels. (**b**) $\delta t_{0}$ as a function of $R_{n}$ for representative values of *R* with 1/τ (orange dashed), 20/τ (green dashed), and 100/τ (red dashed). The optimal ranging precision (black solid) and corresponding optimal photon flux rate $R_{\mathrm{opt}}$ (black dotted) for varying $R_{n}$ are also plotted. Points A, B, and C in both subfigures mark the optimal ranging performance points for different $R_{n}$ values.

Figure 4a further illustrates the variation of ranging precision $\delta t_{0}$ as a function of *R* under different background noise flux rate $R_{n}$, with ToF $t_{0}=10\tau$ fixed. It can be observed that as $R_{n}$ increases, the lower bound $\delta_{R}t_{0}$ (dashed lines) generally increases, indicating that the information available for estimating $t_{0}$ alone reduces due to the increased background noise. On the other hand, with increasing $R_{n}$, the correlation coefficient ρ decreases overall, implying a mitigation of pile-up effects and thus a weakened statistical correlation between $t_{0}$ and *R*. Intuitively, the pile-up effect of the laser pulse manifests as a high count rate at the rising edge, causing the latter half of the pulse to almost entirely fall within the detector dead time. The presence of background noise introduces a nonzero probability that the SPAD is already in dead time triggered by background counts when the pulse arrives, thereby allowing the detector to recover just in time to respond to the latter half of the pulse, alleviating the pile-up effect. As a result of the combined influence of $\delta_{R}t_{0}$ and ρ, the system’s ranging precision $\delta t_{0}$ at lower *R* deteriorates with increasing $R_{n}$, whereas at higher *R* it actually improves. Figure 4b shows the dependence of $\delta t_{0}$ on $R_{n}$ at two fixed detection rates with R=1/τ and R=100/τ, more clearly demonstrating this trend.

Figure 4b also depicts the system’s optimal ranging precision and the corresponding optimal received photon flux rate $R_{\mathrm{opt}}$ as functions of $R_{n}$. It can be seen that, while the optimal ranging precision remains approximately constant with increasing $R_{n}$ (around 1.6/τ to 2.1/τ), the optimal detection rate $R_{\mathrm{opt}}$ undergoes two abrupt jumps near $R_{n}\approx 0.01/\tau$ and 0.06/τ, increasing first from about 1/τ to 20/τ and then from 20/τ to roughly 80/τ, indicating a significant increase in the required photon flux. These jumps occur because the CRLB curve develops new minima around R≈20/τ at $R_{n}\approx 0.01/\tau$ and R≈100/τ at $R_{n}\approx 0.06/\tau$, as a result of delicate interplay between $\delta_{R}t_{0}$ and $\rho^{2}$ [see points A, B, and C in Figure 4a].

To avoid excessive echo power that could strain the system or exceed safety thresholds (e.g., the eye-safety limit), the ranging performance can be considered under a photon-flux upper bound constraint. For example, Figure 4b presents $\delta t_{0}$ constrained by R<20/τ, corresponding to the optimal detection rate after the first jump. Although the system’s ranging performance degrades beyond the theoretical optimum for $R_{n}>0.06/\tau$, the maximum deterioration is about 30%, which can serve as a practical reference for the trade-off between photon flux and ranging capability in system design.

It should be noted that, in practical applications, the maximum allowable laser output is influenced by multiple factors, as shown in Equation (1) [7,39]. Consequently, each dToF system type requires a separate analysis. For example, consider two otherwise identical dToF systems, A and B, with SPADs having PDEs of 30% and 10%, respectively. To achieve the same *R* value, the required laser output for system B would be three times that of system A. This implies that, while system A may remain within safety limits, system B could exceed them. Therefore, it is difficult to establish a universal laser safety limit in terms of *R*. Nevertheless, as illustrated in Figure 4, the theoretical results derived here show system performance corresponding to different *R* values, i.e., the response when each histogram bin receives different numbers of photons. Based on this key intermediate parameter, system designers can readily evaluate the upper bound of acceptable *R* values.

#### 3.1.2. System Optimization over the Full Range

In the absence of prior knowledge about the target distance, it may seem necessary to evaluate the ranging precision over all possible values of $t_{0}$ to assess overall system performance and enable further optimization. However, the following analysis demonstrates that it suffices to consider only the case where the received laser pulse lies within the first bin, i.e., $t_{0}\in \left[0,\tau \right)$, to generalize the ranging performance over the entire histogram range.

From Equation (21), it is clear that, for a given system and fixed *R*, the variance of the ToF estimate $\delta t_{0}$ is determined solely by the values of $F_{i}$ associated with the bins occupied by the received laser pulse. Moreover, Equation (8) indicates that the values of $F_{i}$ are determined by the value of $F_{l_{1}}$ corresponding to the leading bin occupied by the received laser pulse, the photon detection probabilities $q_{i}$ across the laser pulse, and the background photon triggering probability $q_{b}$. For systems employing a single-TDC architecture, when $t_{0}$ is shifted by an integer number of bins, the ratio $F_{i}/F_{l_{1}}=\prod_{j=l_{1}}^{i-1}p_{j}$ remains invariant. According to Equation (25), since $\rho^{2}$ is homogeneous with respect to $F_{i}$, it remains unchanged as well, and therefore $\delta t_{0}{|}_{t_{0}=\left(l_{1}+\varepsilon \right)\tau }=\sqrt{F_{0}/F_{l_{1}}}\cdot \delta t_{0}{|}_{t_{0}=\varepsilon \tau }$. Equation (8) implies $F_{l_{1}>T}=F_{0}{\left(1-q_{b}\right)}^{l_{1}-T}$, and thus the ranging precision for the single-TDC architecture decreases exponentially with increasing leading bin index $l_{1}$ under a fixed photon flux rate *R*, expressed as

(35) $$\delta t_{0}{|}_{t_{0}=\left(l_{1}+\varepsilon \right)\tau }={\left(1-q_{b}\right)}^{l_{1}-T}\delta t_{0}{|}_{t_{0}=\varepsilon \tau }.$$

For a multi-event TDC architecture, Equation (7) implies that $F_{i\leq l_{1}}=1/\left(1+q_{b}T\right)$, i.e., all *F* values prior to the arrival of the laser pulse are identical, thereby leading to the relation

(36) $$\delta t_{0}{|}_{t_{0}=\left(l_{1}+\varepsilon \right)\tau }=\delta t_{0}{|}_{t_{0}=\varepsilon \tau }.$$

which means under a fixed *R*, the ranging precision remains unchanged when $t_{0}$ is shifted by an integer number of bins for a multi-event TDC architecture. Therefore, the overall ranging capability of the system across the full ToF range can be fully characterized by analyzing $\delta t_{0}$ within the single-bin interval for both single- and multi-event TDC architectures.

**Figure 5:**
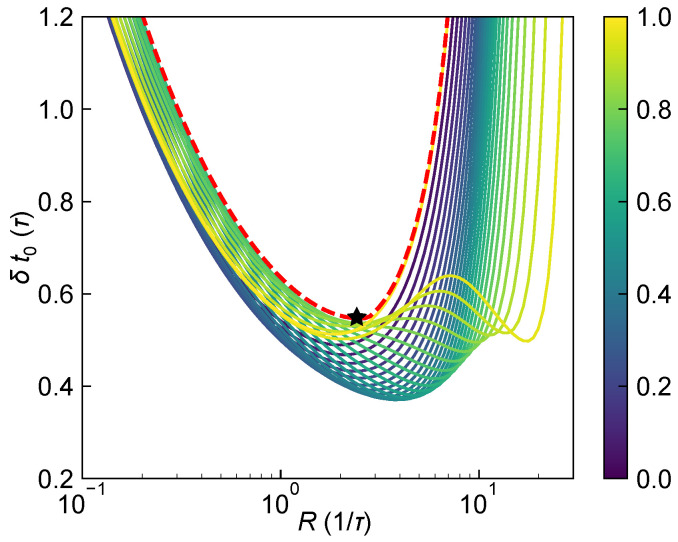
Ranging precision $\delta t_{0}$ as a function of *R* for different values of $t_{0}/\tau$ varying from 0 (purple) to 1 (yellow) with laser pulse width of 0.6τ. The worst-case ranging precision over $t_{0}\in \left[0,\tau \right)$, denoted as $\Delta t_{0}$, is shown as a red dashed line. The minimum standard deviation $\Delta_{m}t_{0}$ and the corresponding optimal received photon flux rate $R_{\mathrm{opt}}$ are marked by a black star.

Figure 5 shows how $\delta t_{0}$ varies with *R* for different values of $t_{0}$ within a single bin [0,τ). It is evident that, while the absolute values differ slightly, the overall trend remains consistent: $\delta t_{0}$ decreases initially with increasing *R*, then increases again. Notably, for $t_{0}=0$ and $t_{0}=\tau$, the curves coincide exactly due to the symmetry introduced by a full-bin shift. Since no prior knowledge of the target ToF is available, i.e., $t_{0}$ can take any value within [0,τ), to ensure the system’s ranging performance under the worst-case scenario, the system’s ranging performance at a given *R* is defined as the maximum value of $\delta t_{0}$ over $t_{0}\in \left[0,\tau \right)$, denoted as

(37) $$\Delta t_{0}=\max_{\varepsilon \in [0,1)}\left\{\delta t_{0}{|}_{t_{0}=\varepsilon \tau }\right\},$$

corresponding to the upper envelope in Figure 5 (red dashed line). It can be observed that $\Delta t_{0}$ at different values of *R* corresponds to the $\delta t_{0}$ over different $t_{0}\in \left[0,\tau \right)$, making it difficult to derive a closed-form expression, and thus must be obtained numerically. In practical systems, by tuning the laser power to adjust *R*, the system can be operated at the minimum of $\Delta t_{0}$, denoted as $\Delta_{m}t_{0}$, and the corresponding optimal photon arrival rate $R_{\mathrm{opt}}$, as marked by the black star in the figure. This point can be regarded as the fundamental ranging precision limit of the system under complete lack of prior knowledge about the target.

**Figure 6:**
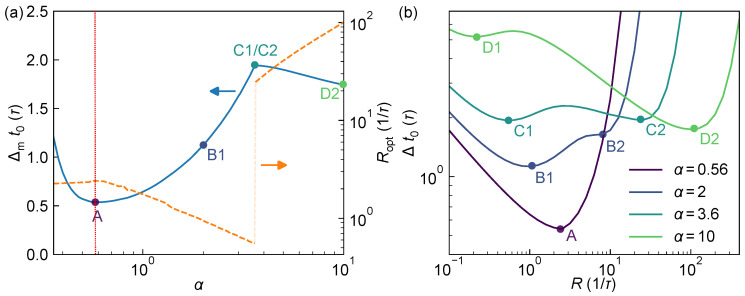
Ranging performance of the system as a function of laser pulse width w=ατ. (**a**) Theoretical optimal ranging precision $\Delta_{m}t_{0}$ (solid blue, left axis) and the corresponding optimal detection rate $R_{\mathrm{opt}}$ (dashed orange, right axis) for different α. The translucent section of the $R_{\mathrm{opt}}$ curve indicates the region of abrupt transition. Points A–D correspond to the minima under α=0.56, 2, 3.6, and 10, respectively, and multiple local minima are labeled as 1 and 2 for distinction. The optimal operating points is indicated by the red dotted line. (**b**) Ranging precision $\Delta t_{0}$ as a function of *R* for the four representative pulse widths in (**a**), along with the respective optimal points A–D.

One of the key design parameters in a dToF system is the ratio between the laser pulse width *w* and the TDC resolution τ. Figure 6a illustrates the system’s optimal ranging performance $\Delta_{m}t_{0}$ (normalized to τ) as a function of laser pulse width w=ατ under a fixed τ. As α approaches zero, the laser pulse energy becomes almost entirely concentrated within a single bin, and the histogram fails to capture sufficient information to support sub-bin precision, which results in the divergence of the ranging variance. As α increases, more timing information becomes available from the histogram, and the ranging precision improves. The optimal performance is achieved at α≈0.56, where $\Delta_{m}t_{0}$ reaches a minimum of approximately 0.53τ at $R_{\mathrm{opt}}\approx 2.4/\tau$. In practical terms, for a TDC resolution of 1 ns, a single pulse measurement under a laser pulse with width of 560 ps corresponds to a rannging standard deviation of about 300 ps, or roughly 45 mm, theoretically, and can be futher improved to 4.5 mm on the accumulated histogram from 100 pulses. As α increases beyond this point, $\Delta_{m}t_{0}$ gradually worsens. However, as shown in Figure 6b, a secondary minimum emerges (B2) at higher *R* (at R≈7/τ), and this second minimum decreases as α increases. When α≈3.64, both minima (C1 and C2) converge to approximately 1.90. With further increases in α, the secondary minimum (D2) becomes the global minimum, yielding improved system performance again. Nevertheless, the corresponding optimal $R_{\mathrm{opt}}$ jumps from around 2/τ to approximately 30/τ, implying a more than tenfold increase in required laser power, which may impose significantly higher demands on the laser emission capability of the dToF system. Therefore, in practical dToF system design, the pulse width should be kept with an ideal value near 0.56τ to achieve the best overall ranging precision. Furthermore, since the system parameter *R* may inevitably fluctuate around $R_{\mathrm{opt}}$ in practice, potentially degrading the ranging performance, we also investigated the sensitivity of the optimal performance to such variations [see Section S9 of the Supplementary Information (SI)]. The analysis shows that, even when *R* deviates from $R_{\mathrm{opt}}$ by as much as ±20%, the resulting performance loss remains limited to about 1%.

**Figure 7:**
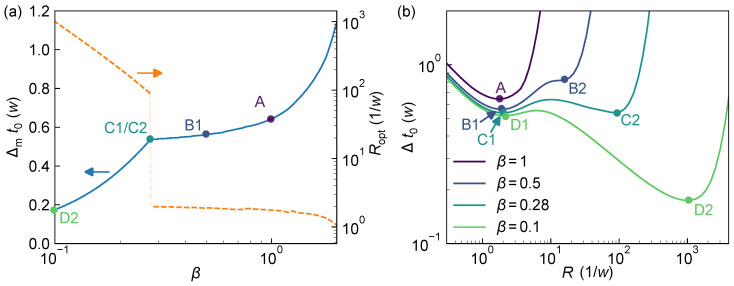
System ranging performance bound as a function of TDC resolution τ=βw. (**a**) Theoretical optimal ranging precision $\Delta_{m}t_{0}$ (solid blue, left axis) and the corresponding optimal photon rate $R_{\mathrm{opt}}$ (dashed orange, right axis) under varying β. The translucent section of the $R_{\mathrm{opt}}$ curve indicates the region of abrupt transition. Points A–D correspond to the minima under β=1, 0.5, 0.28, and 0.1, respectively, and multiple local minima are labeled as 1 and 2 for distinction. (**b**) $\Delta t_{0}$ as a function of *R* at representative values of β, along with their optimal performance points A–D.

When the laser pulse width *w* is fixed, Figure 7a illustrates the system’s ranging performance $\Delta_{m}t_{0}$ (normalized to *w*) and the corresponding optimal detection rate $R_{\mathrm{opt}}$ under different TDC resolutions τ=βw. For large β, the histogram bin width exceeds the laser pulse width, making it difficult to extract sufficient timing information from the histogram for sub-bin accuracy. As a result, $\Delta_{m}t_{0}$ tends toward infinity. As β decreases, temporal resolution of the pulse improves, leading to a steady decrease in $\Delta_{m}t_{0}$. During this regime, although $R_{\mathrm{opt}}$ increases, it remains within a moderate range (typically between 1/τ and 2/τ). Moreover, when β<1, the curve of $\Delta_{m}t_{0}$ begins to flatten, indicating diminishing returns from further improvements in TDC resolution. For example, improving TDC resolution from β=0.6 to β=0.3 (i.e., halving τ) reduces $\Delta_{m}t_{0}$ from 0.57w to about 0.54w, an improvement of only ~5%.

On the other hand, Figure 7b shows that, at larger *R* values ($R_{\mathrm{opt}}>100/w$), an alternative local minimum (B2) in $\Delta t_{0}$ emerges and continues to decrease with smaller β. This behavior resembles that observed in Figure 6, which is reasonable since increasing τ with fixed *w* is equivalent to decreasing *w* with fixed τ. At around β≈0.28, the larger-*R* local minimum (C2) becomes the global minimum and continues to drop rapidly as β decreases. As β approaches 0, the system effectively samples the return pulse in a nearly continuous manner, allowing $\Delta_{m}t_{0}$ to approach zero. However, since the bin width τ also tends toward zero in this limit, achieving meaningful photon counts per bin would require an infinite photon flux rate, which is reflected in the divergence of $R_{\mathrm{opt}}$, and is clearly impractical for real-world applications.

Therefore, when the laser pulse width *w* is fixed, a TDC resolution slightly smaller than *w* is sufficient to achieve near-optimal system performance without requiring excessively high *R*. Further reducing τ below 0.3w may yield marginal theoretical gains in precision, but these come at the cost of rapidly increasing photon demands and greater susceptibility to practical limitations such as TDC nonlinearity [16] and timing jitter [40,41], making the theoretical optimum difficult to attain in practice.

### 3.2. Ranging Performance for *s*-PNR SPAD

**Figure 8:**
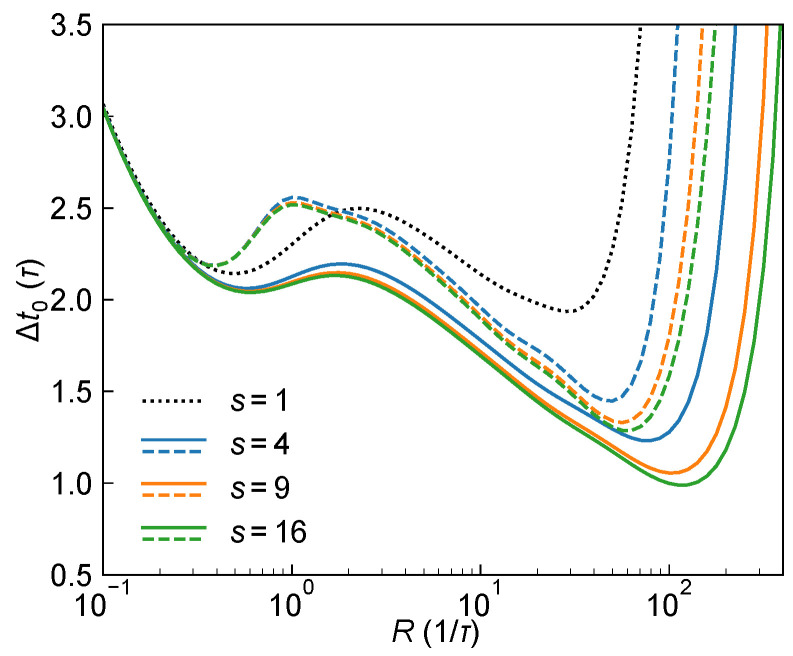
Ranging precision $\Delta t_{0}$ versus recived photon flux rate *R* for 1-PNR SPAD (black dotted), 4-PNR (blue), 9-PNR (orange), and 16-PNR (green) detectors under Type I (solid) and Type II (dashed) systems.

Figure 8 illustrates the theoretical optimal ranging precision $\Delta t_{0}$ as a function of the photon rate *R*, using 4-PNR (e.g., 2×2 subpixels), 9-PNR (e.g., 3×3), and 16-PNR SPAD (e.g., 4×4) macro-pixels under both Type I and Type II system architectures, with the laser pulse width set to w=2τ. Compared to the 1-PNR case, increasing the number of subpixels, i.e., improving photon-number resolution, shifts the $\Delta_{m}t_{0}$ curves downward and to the right, indicating improved theoretical performance and a higher required return photon rate *R*. Moreover, as the number of subpixels *s* increases from 1 to 16, the overall shift of the curves gradually diminishes, indicating diminishing returns in performance improvement. In addition, for a fixed *s*, Type II system consistently yields worse $\Delta t_{0}$ than Type I, which is consistent with our previous analysis in Equation (34): the lack of TDC trigger count information in Type II leads to stronger inter-bin correlations due to dead time, increasing the covariance and reducing the overall Fisher information.

**Figure 9:**
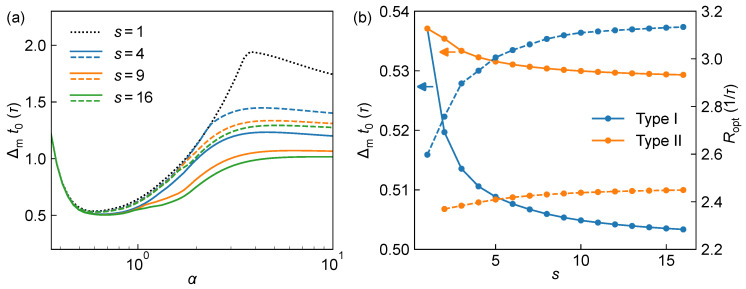
System ranging performance as a function of the number of subpixels *s*. (**a**) Theoretical optimal ranging precision $\Delta_{m}t_{0}$ versus normalized laser pulse width α for 1- (black dotted), 4- (blue), 9- (orange), and 16-PNR (green) detectors under Type I (solid) and Type II (dashed) architectures. (**b**) $\Delta_{m}t_{0}$ (solid, left axis) and the corresponding optimal photon rate $R_{\mathrm{opt}}$ (dashed, right axis) as functions of subpixel number *s* for Type I (blue) and Type II (orange) architectures.

Figure 9a further shows the theoretical optimal ranging precision $\Delta_{m}t_{0}$ and the corresponding optimal photon flux rate $R_{\mathrm{opt}}$ as functions of the normalized pulse width α=w/τ for various values of *s*, under Type I and II systems. When α is large, the required $R_{\mathrm{opt}}$ is also high, and increasing *s* leads to a clear improvement in ranging precision, with Type I consistently outperforming Type II. In contrast, for α<1, $R_{\mathrm{opt}}$ remains low, and the benefit of higher photon-number resolution becomes marginal, as evidenced by the nearly overlapping $\Delta_{m}t_{0}$ curves across different values of *s*. Figure 9b shows the minimal $\Delta_{m}t_{0}$ achieved at the optimal α for each *s*, which reflects the improvement in the theoretical ranging performance limit enabled by PNR capability. For Type I configuration, increasing *s* from 1 to 16 improves $\Delta_{m}t_{0}$ from approximately 0.536τ to 0.503τ—an improvement of less than 10%. The marginal benefit quickly decreases: increasing *s* from 1 to 9 yields a ∼9.4% improvement, but going from 9 to 16 only adds ∼0.5%. For Type II configuration, the gains are even smaller: increasing *s* from 1 to 16 improves $\Delta_{m}t_{0}$ by only ∼1.3%, from ∼0.536τ to ∼0.529τ. The same figure also plots the optimal photon rate $R_{\mathrm{opt}}$ versus *s*. Although $R_{\mathrm{opt}}$ does increase with *s*, it remains in the modest range of 2∼3, indicating that the high dynamic range potential of large-*s* PNR SPADs is not fully utilized, which further limits the performance benefits brought by additional subpixels.

In summary, while s-PNR SPAD detectors can effectively increase the system’s dynamic range and maintain good performance under high photon flux, the theoretical optimal ranging precision limit is typically reached under conditions of relatively narrow laser pulse widths and moderate photon flux (on the order of 1 photons per pulse). In this regime, even an s-PNR detector triggers only a few subpixels on average, so the multi-pixel advantage is not fully utilized. This explains why the improvement in the ideal precision limit compared with a 1-PNR detector appears limited. Moreover, practical issues such as inter-subpixel mismatch and optical/electrical crosstalk across subpixels may degrade overall macro-pixel performance as *s* increases [15], highlighting the importance of carefully balancing design trade-offs in system implementation.

### 3.3. Case Study: Selection of the Optimal Operating Point with Varying Ambient Light

The CRLB formulas derived above provide a comprehensive framework for evaluating the ranging performance of dToF systems. Here, a representative application scenario is considered to illustrate how these theoretical results can guide practical system operation.

Consider the following classic scenario: as the ambient light perceived by the dToF system increases, how should the system adjust to maintain ranging accuracy? Should the system employ adjustable attenuation (e.g., via a tunable filter or variable-aperture optics) to simultaneously reduce both ambient and signal photon flux, should it increase the signal photon flux, or should both strategies be applied concurrently?

**Figure 10:**
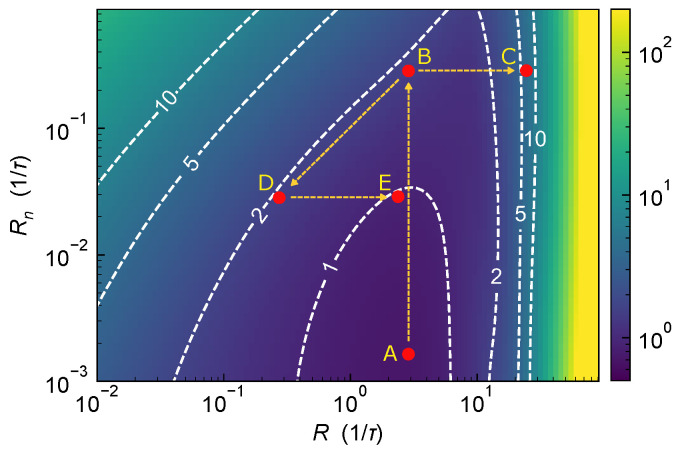
Illustration of $\Delta t_{0}$ under different peak received photon flux rate *R* and background noise flux rate $R_{n}$ for a laser pulse width of 0.56τ. The optimal working point under low ambient light is denoted as A. When ambient light increases, the system moves to point B. Increasing laser power alone shifts the working point to C. Applying attenuation moves the system along a $\Delta t_{0}$ contour to D. A combined strategy of attenuation followed by laser power increase shifts the system to E.

Figure 10 shows the $\Delta t_{0}$ under different peak received photon flux rate *R* and background noise flux rate $R_{n}$ for a laser pulse width of 0.56τ (corresponding to the optimal pulse width identified in Figure 6). Initially, assume the system operates indoors or at night, where $R_{n}$ is low (approximately $10^{-3}/\tau$). By adjusting *R* to operate at the optimal working point A, the corresponding ranging precision is $\Delta t_{0}\approx 0.53\tau$. Now consider the system being deployed outdoors or from nighttime to daytime conditions, resulting in increased ambient light, $R_{n}>0.1/\tau$. In this case, the system working point shifts from A to B, and the ranging precision degrades to $\Delta t_{0}\approx 2\tau$. How should the system be adjusted to restore its ranging performance? As shown in the Figure 10, increasing the laser output power to improve the signal-to-noise ratio moves the working point to the right (e.g., to C), but this actually worsens the ranging precision. This occurs because, with a relatively narrow laser pulse, higher return photon flux amplifies the pile-up effect, further degrading system performance. Alternatively, applying attenuation to simultaneously reduce both $R_{n}$ and *R* moves the working point along a 45° direction toward the lower-left (due to the log-log scale with equal axis ratios), e.g., to D. However, the ranging precision remains nearly constant along this trajectory, corresponding to movement along a $\Delta t_{0}$ contour, and therefore the system performance is not improved.

Importantly, Figure 10 shows that, after adding attenuation and adjusting the working point to D, a subsequent increase in laser output power can move the system to point E. At this new operating point, the ranging precision improves to approximately $\Delta t_{0}\approx \tau$, effectively doubling the performance compared to the original point B. This case study illustrates how the performance bounds derived in this work can guide practical system operation.

### 3.4. Section Summary

In this section, based on the CRLB derived in Section 2, the ranging performance of the system was analyzed at fixed distances, across the full range, and under *s*-PNR detection. The main conclusions are as follows:

(1) The pile-up effect caused by dead time and the resulting distortion of the histogram relative to the laser pulse lead to coupling between the unknown parameters $t_{0}$ and *R*. Consequently, there exists an optimal *R* that minimizes the estimation standard deviation. And as *R* approaches infinity, nearly all histogram counts occur in the first bin, preventing sub-bin resolution and causing the standard deviation of $t_{0}$ to diverge.
(2) In the presence of ambient light, although the additional counts reduce the available signal counts and hence the effective information, at large *R*, ambient light mitigates the pile-up effect through dead-time-induced saturation. This reduces the coupling between *R* and $t_{0}$, effectively improving the ranging performance.
(3) Considering the full range of distances, for Gaussian-shaped laser pulses with a fixed TDC resolution τ, the optimal pulse FWHM is approximately 0.56τ, yielding a best achievable ranging precision of about $0.53\tau /\sqrt{N}$, with a corresponding optimal received photon flux $R_{\mathrm{opt}}\approx 2.4/\tau$. Conversely, for a fixed laser pulse width *w*, the optimal TDC resolution is slightly larger than 0.28w, yielding a best precision of approximately $0.54w/\sqrt{N}$ and $R_{\mathrm{opt}}\approx 2/w$.
(4) For *s*-PNR systems, increasing *s* improves the overall ranging precision at high *R*, indicating an increased dynamic range and enhanced capability under high photon flux conditions. However, for the system’s theoretical optimal precision, the best laser pulse FWHM remains near 0.56τ for a given TDC resolution, with the optimal photon flux $R_{\mathrm{opt}}$ in the range of 2/τ∼3/τ. In this scenario, the multi-pixel advantage of *s*-PNR detectors is not fully exploited, limiting their improvement of the system’s ranging performance.
(5) Finally, a case study under strong ambient light demonstrates how the derived CRLB can guide practical system operation, highlighting the relevance of the theoretical framework for real-world system design and optimization.

## 4. Conclusions

In conclusion, this work theoretically derives the Cramér–Rao lower bound for the ranging performance of dToF LiDAR systems considering the effect of dead time, and further extends the analysis to SPADs with photon-number-resolving capabilities. The theoretical results agree well with MC simulations, validating the correctness of the derivation. According to the analysis, the pile-up effect caused by dead time not only reduces the information content of each histogram bin, but also introduces coupling between the time-of-flight and the received photon flux rate, further degrading the ranging performance. Because of this coupling, the received photon flux rate exhibits an optimal value rather than the traditionally assumed monotonic performance improvement with increasing photon flux; moreover, ambient background light can, under certain conditions, enhance system performance. Further analysis is conducted to determine the optimal laser pulse width and TDC resolution for achieving the theoretical ranging limit. For SPADs with subpixels, the theoretical analysis shows that dToF systems should record TDC trigger events to fully exploit the information gain provided by photon-number-resolving capability, thereby achieving the theoretical optimal ranging performance. However, while photon-number resolution can improve the system’s dynamic range, its impact on the theoretical limit of ranging precision is quite limited.

It is worth noting the limitations of the present study. First, our analysis is based solely on numerical simulations, and experimental validation of the proposed models is still required to fully confirm their accuracy in practical scenarios. Second, although the performance bounds for *s*-PNR detectors have been provided, these bounds remain relatively loose for asynchronously triggered *s*-PNR detectors operating under strong ambient light. A more detailed theoretical analysis to obtain tighter bounds would be desirable. Future research could extend this study by incorporating experimental measurements and investigating tighter bounds for asynchronously triggered *s*-PNR detectors, which would provide a more comprehensive understanding of system performance under real-world conditions.

We believe the theoretical results derived here represent the fundamental lower bound of ranging performance for dToF systems and can serve as valuable guidance for system design and optimal operating point selection.

## Data Availability

Data is contained within the article.

## Supplementary Materials

The following supporting information can be downloaded at: https://www.mdpi.com/article/10.3390/s25196184/s1, Section S1: Effects for Unequal Dead Times of TDC and SPAD; Section S2: Effects of Afterpulsing; Section S3: Effects for Crosstalk; Section S4: Effects of Timing Jitter; Section S5: Asynchronous Triggering of *s*-PNR Detectors; Section S6: Error Analysis under the Large-*N* Approximation in Type II Systems; Section S7: Comparison with the Coates Algorithm; Section S8: Performance Bounds for an Asymmetric Gaussian Pulse; Section S9: Sensitivity analysis of the theoretical ranging precision limit with respect to *R*.

## Abbreviations

The following abbreviations are used in this manuscript:

ToF	Time-of-flight
dToF	Direct time-of-flight
SPAD	Single-photon avalanche diode
CRLB	Cramér–Rao lower bound
LiDAR	Light detection and ranging
TDC	Time-to-digital converter
MC	Monte Carlo
MLE	Maximum likelihood estimation
PDE	Photon detection efficiency
*s*-PNR	*s*-photon-number resolution

## Glossory

The following symbols are used in this manuscript:

T	system dead time
f(t)	waveform of the emitted laser pulse
*R*	peak photon flux rate absorbed by the SPAD
$\eta_{s}$	the PDE of SPAD
$\eta_{o}$	the overall optical transmittance of the system
*r*	the target reflectivity
*D*	the diameter of the receiving lens
*z*	the target distance
*c*	speed of light
$t_{0}$	round-trip ToF
$S_{i}$	mean photon count of the signal within the time interval of the *i*-th histogram bin

τ	bin width of the histogram
$R_{n}$	average photon flux of noise absorbed by the SPAD
*b*	average photon count of noise
$p_{i}$	the probability of no detection event occurring in the *i*-th bin
$q_{i}$	the probability of detection event occurring in the *i*-th bin
h	histogram
h(i)	the random variable of the count in the *i*-th bin of the histogram
$h_{i}$	the specific value of the count in the *i*-th bin of the histogram

*T*	the number of histogram bins occupied by dead time
B	binomial distribution
*N*	the number of the independent pulse measurements
$N_{i}^{\prime }$	the reduced number of the pulse measurements for the *i*-th bin
$H_{i}$	the conditional probability of the random variable h(i)
$Q_{i}$	the expected count in the *i*-th bin for a single measurement
$F_{i}$	the reduced factor of measurable pulses due to dead time
$q_{b}$	the probability of a trigger in one bin under background noise without accounting
	for dead time effect
*A*	probability of a “negative bin” not affected by dead time
m	the counts of “negative bins”
*s*	the number of subpixels
$R^{\prime }$	peak photon flux rate absorbed by a subpixel
$v_{i}$	the probability of no detection event occurring in the *i*-th bin for a subpixel
$u_{i}$	the probability of detection event occurring in the *i*-th bin for a subpixel
k(i)	the random variable of the count in the *i*-th bin of the histogram (the number
	of the subpixels triggered)
$K_{i}$	the conditional probability of the random variable k(i)
$J_{s}\left(h_{i},k_{i}\right)$	the number of ways to allocate $k_{i}$ triggered subpixels across $h_{i}$ TDC events
θ	the unknows parameters to be estimated
$I_{ij}$	the Fisher information matrix
$I_{ij}$	the Fisher information corresponding to a single pulse measurement
$\hat{t_{0}}$	estimator of $t_{0}$
$\rho^{2}$	the correlation coefficient between $t_{0}$ and *R*
$C_{ij}$	the covariance between k(i) and k(j) normalized by *s* and *N*
Y(i)	the random variable of SPADs triggered in a single detection event at the *i*-th bin
*w*	FWHM of the Gaussian-shaped laser pulse
$R_{\mathrm{opt}}$	the optimal *R* corresponding to the lower bound of the standard deviation of the esminated $t_{0}$
$\delta t_{0}$	the lower bound of the standard deviation of the ToF measurement per single pulse
	measurement at given $t_{0}$ and *R*
$\delta_{R}t_{0}$	the lower bound of the standard deviation of the ToF measurement per single pulse
	measurement at given $t_{0}$ assuming a known *R*
$\Delta t_{0}$	the upper bound (worst case) of $\delta t_{0}$ at given *R* over varying $t_{0}$
$\Delta_{m}t_{0}$	the lower bound of $\Delta t_{0}$ over varying *R*

## Appendix A. Derivation Details of the Steady State

The joint probability distribution for an arbitrary continuous span of T+1 “negative bins” before the measurement cycle starts follows a multinomial distribution:

(A1)  P{h(j)=hj,h(j+1)=hj+1,…,h(j+T)=hj+T} =Nhjhj+1…hj+Tqb1+qbT∑k=jj+Thk1−qb1+qbTN−∑k=jj+Thk,

where (Nhjhj+1…hj+T) is the multinomial coefficient

(A2) Nhjhj+1…hj+T=N!hj!hj+1!…hj+T!(N−∑k=0Thk)!.

And the expected histogram values for the virtual bins is

(A3)  E[h(i<0)] =∑mmiP{h(−1)=m1,h(−2)=m2,…,h(−T−1)=mT+1} =11+qbTN∑mmiNm0…mTqbmT′(1−qb)N−mT′ =11+qbTN∂[qbT+qbx+(1−qb)]N∂xx=1 =Nqb1+qbT,

where $m=(m_{1},\ldots ,m_{T})$ and $m_{T}^{\prime }=\sum_{i=1}^{T+1}m_{i}$.

## Appendix B. Derivation Details of the Fisher Information

The detailed derivation of the Fisher information is presented below.

For Equation (18):

(A4) $$\begin{array}{ccc} & & \sum_{h_{l}}{\tilde{H}}_{l-1}\frac{1}{H_{l}}\frac{\partial H_{l}}{\partial \theta_{i}}\frac{\partial H_{l}}{\partial \theta_{j}}\\ & = & \sum_{h_{l}}{\tilde{H}}_{l-1}H_{l}{\left(\frac{h_{l}-N_{l}^{\prime }q_{l}}{p_{l}q_{l}}\right)}^{2}\frac{\partial q_{l}}{\partial \theta_{i}}\frac{\partial q_{l}}{\partial \theta_{j}}\\ & = & \sum_{h_{l-1}}{\tilde{H}}_{l-1}\frac{1}{p_{l}^{2}q_{l}^{2}}\frac{\partial q_{l}}{\partial \theta_{i}}\frac{\partial q_{l}}{\partial \theta_{j}}\sum_{h_{l}}H_{l}{\left(h_{l}-N_{l}^{\prime }q_{l}\right)}^{2}\\ & = & \sum_{h_{l-1}}{\tilde{H}}_{l-1}\frac{1}{p_{l}^{2}q_{l}^{2}}\frac{\partial q_{l}}{\partial \theta_{i}}\frac{\partial q_{l}}{\partial \theta_{j}}D\left[H_{l}\right]\\ & = & \sum_{h_{l-1}}{\tilde{H}}_{l-1}\frac{1}{p_{l}^{2}q_{l}^{2}}\frac{\partial q_{l}}{\partial \theta_{i}}\frac{\partial q_{l}}{\partial \theta_{j}}N_{l}^{\prime }q_{l}p_{l}\\ & = & \sum_{h_{l-1}}{\tilde{H}}_{l-1}\frac{N_{l}^{\prime }}{p_{l}q_{l}}\frac{\partial q_{l}}{\partial \theta_{i}}\frac{\partial q_{l}}{\partial \theta_{j}}\\ & = & \frac{1}{p_{l}q_{l}}\frac{\partial q_{l}}{\partial \theta_{i}}\frac{\partial q_{l}}{\partial \theta_{j}}\sum_{h_{l-1}}{\tilde{H}}_{l-1}N_{l}^{\prime }\\ & = & \frac{E[h(l)]}{q_{l}}\frac{1}{p_{l}q_{l}}\frac{\partial q_{l}}{\partial \theta_{i}}\frac{\partial q_{l}}{\partial \theta_{j}}\\ & = & N\frac{F_{l}}{p_{l}q_{l}}\frac{\partial q_{l}}{\partial \theta_{i}}\frac{\partial q_{l}}{\partial \theta_{j}}. \end{array}$$

For the third term of Equation (17):

(A5) $$\begin{array}{ccc} & & \sum_{h_{l}}\frac{\partial {\tilde{H}}_{l-1}}{\partial \theta_{i}}\frac{\partial H_{l}}{\partial \theta_{j}}\\ & = & \lim_{\begin{array}{c} \Delta_{i}\to 0\\ \Delta_{j}\to 0 \end{array}}\sum_{h_{l}}\left(\frac{{\tilde{H}}_{l-1}\left(\theta_{i}+\Delta_{i}\right)-{\tilde{H}}_{l-1}\left(\theta_{i}\right)}{\Delta_{i}}\right)\left(\frac{H_{l}\left(\theta_{j}+\Delta_{j}\right)-H_{l}\left(\theta_{j}\right)}{\Delta_{j}}\right)\\ & = & \lim_{\begin{array}{c} \Delta_{i}\to 0\\ \Delta_{j}\to 0 \end{array}}\sum_{h_{l}}\frac{{\tilde{H}}_{l-1}\left(\theta_{i}+\Delta_{i}\right)H_{l}\left(\theta_{j}+\Delta_{j}\right)-{\tilde{H}}_{l-1}\left(\theta_{i}+\Delta_{i}\right)H_{l}\left(\theta_{j}\right)-{\tilde{H}}_{l-1}\left(\theta_{i}\right)H_{l}\left(\theta_{j}+\Delta_{j}\right)+{\tilde{H}}_{l-1}\left(\theta_{i}\right)H_{l}\left(\theta_{j}\right)}{\Delta_{i}\Delta_{j}}. \end{array}$$

For each $\sum_{h_{l}}{\tilde{H}}_{l-1}\left(\cdot \right)H_{l}\left(\cdot \right)=1$, so the above equation is identical to 0.

For Equation (29):

(A6) $$\begin{array}{ccc} & & \sum_{k_{l},h_{l}}{\tilde{K}}_{l-1}\frac{1}{K_{l}}\frac{\partial K_{l}}{\partial \theta_{i}}\frac{\partial K_{l}}{\partial \theta_{j}}\\ & = & \sum_{k_{l},h_{l}}{\tilde{K}}_{l-1}K_{l}{\left(\frac{k_{l}-sN_{l}^{\prime }u_{l}}{v_{l}u_{l}}\right)}^{2}\frac{\partial u_{l}}{\partial \theta_{i}}\frac{\partial u_{l}}{\partial \theta_{j}}\\ & = & \sum_{k_{l-1},h_{l-1}}{\tilde{K}}_{l-1}\frac{1}{v_{l}^{2}u_{l}^{2}}\frac{\partial u_{l}}{\partial \theta_{i}}\frac{\partial u_{l}}{\partial \theta_{j}}\sum_{k_{l},h_{l}}K_{l}{\left(k_{l}-sN_{l}^{\prime }q_{l}\right)}^{2}\\ & = & \sum_{k_{l-1},h_{l-1}}{\tilde{K}}_{l-1}\frac{1}{v_{l}^{2}u_{l}^{2}}\frac{\partial u_{l}}{\partial \theta_{i}}\frac{\partial u_{l}}{\partial \theta_{j}}D\left[K_{l}|N_{l}^{\prime }\right]\\ & = & \sum_{k_{l-1},h_{l-1}}{\tilde{K}}_{l-1}\frac{1}{v_{l}^{2}u_{l}^{2}}\frac{\partial u_{l}}{\partial \theta_{i}}\frac{\partial u_{l}}{\partial \theta_{j}}sN_{l}^{\prime }u_{l}v_{l}\\ & = & \sum_{k_{l-1},h_{l-1}}{\tilde{K}}_{l-1}\frac{sN_{l}^{\prime }}{v_{l}u_{l}}\frac{\partial u_{l}}{\partial \theta_{i}}\frac{\partial u_{l}}{\partial \theta_{j}}\\ & = & \frac{s}{v_{l}u_{l}}\frac{\partial u_{l}}{\partial \theta_{i}}\frac{\partial u_{l}}{\partial \theta_{j}}\sum_{k_{l-1},h_{l-1}}{\tilde{K}}_{l-1}N_{l}^{\prime }\\ & = & \frac{E[k(l)]}{u_{l}}\frac{s}{v_{l}u_{l}}\frac{\partial u_{l}}{\partial \theta_{i}}\frac{\partial u_{l}}{\partial \theta_{j}}\\ & = & sN\frac{F_{l}}{v_{l}u_{l}}\frac{\partial u_{l}}{\partial \theta_{i}}\frac{\partial u_{l}}{\partial \theta_{j}}. \end{array}$$

## Appendix C. Inverse of the Covariance Matrix C(*θ*)

Denoting diagnal matrix D with element of $D_{ii}=su_{i}F_{i}\left[\left(1-u_{i}\right)+su_{i}\right]$ and column vector ν with elements of $\nu_{i}=u_{i}F_{i}$, matrix C can be rewritten as

(A7) $$C=D-s^{2}\nu \nu^{T}$$

According to the Sherman–Morrison equation [42], $C^{-1}$ can be expressed as

(A8) $$C^{-1}=D^{-1}+\frac{D^{-1}UD^{-1}}{1-\mathrm{tr}(UD^{-1})}.$$

where matrix $U=s^{2}\nu \nu^{T}$. For $\mathrm{tr}\left(UD^{-1}\right)=s^{2}\nu^{T}D^{-1}\nu$, it becomes

(A9) $$C^{-1}=D^{-1}+\frac{s^{2}D^{-1}\nu \nu^{T}D^{-1}}{1-s^{2}\nu^{T}D^{-1}\nu }.$$

## Appendix D. Numerical Simulation

### Appendix D.1. Histogram Generation Based on the Monte Carlo Method

For a 1-PNR SPAD detector, the Monte Carlo procedure for generating a histogram involves the following steps:

(1) Given the TDC resolution τ, laser pulse shape f(t), ToF $t_{0}$, peak received photon rate *R*, and background photon rate $R_{n}$, compute the probability of photon detection in each histogram bin $q_{i}$ according to the Poisson distribution.
(2) Assume the histogram consists of *L* bins and *N* laser pulses are emitted. Generate a set of uniformly distributed random numbers $x_{i}$ in the range [0,1] of length L×N. A photon detection event is recorded in bin *i* if $x_{i}<q_{i}$.
(3) Starting from the first detection event, discard all subsequent detections that occur within a dead time *T* of a previous event.
(4) For the remaining detection events, take the bin index modulo *L* to obtain the ToF, and count their occurrences to generate the final histogram.

By choosing a sufficiently large *L*, the above approach concatenates the *N* acquisition periods into a single continuous sequence, thereby directly capturing dead time effects across pulse periods and more accurately reflecting the actual measurement behavior.

For an *s*-PNR SPAD, the process is similar, except in step 2, the number of subpixel triggers per bin is sampled from a binomial distribution $B(s,u_{i})$, and only bins with at least one triggered subpixel are retained. In step 4, both the TDC-triggered histogram *h* based on detection timing and the actual histogram *k* by summing the number of subpixel triggers corresponding to each ToF are both recorded to generating final histograms.

### Appendix D.2. Parameter Estimation Based on the Maximum Likelihood Method

The maximum likelihood method is used to estimate $t_{0}$ and *R* simultaneously from generated histograms using Monte Carlo simulation. For 1-PNR and Type-I *s*-PNR dToF systems, where the gradient of the log-likelihood function can be derived analytically, a standard gradient-based optimization method is adopted to maximize the log-likelihood function. For Type-II *s*-PNR dToF system, due to the complexity of the gradient expression, a gradient-free optimzation method is used.

For 1-PNR SPAD systems, the log-likelihood function can be derived from Equation (13) as follows:

(A10) $$l\left(h_{l};\theta \right)=\ln P(h_{l},\theta )=\sum_{i=1}^{l}h_{i}\ln q_{i}+\left(N^{\prime }-h_{i}\right)\ln p_{i},$$

and its gradient is derived as

(A11) $$\nabla l\left(h_{l};\theta \right)=\sum_{i=1}^{l}\frac{h_{i}-N^{\prime }q_{i}}{q_{i}}\nabla S_{i}$$

For type I *s*-PNR SPAD systems, the log-likelihood function can be derived from Equation (26) as follows:

(A12) $$l\left(k_{l},h_{l};\theta \right)=\sum_{i=1}^{l}k_{i}\ln u_{i}+\left(sN^{\prime }-k_{i}\right)\ln v_{i},$$

and the gradient is derived as

(A13) $$\nabla l\left(h_{l};\theta \right)=\sum_{i=1}^{l}\frac{k_{i}-sN^{\prime }u_{i}}{u_{i}}\nabla S_{i}.$$

For type II *s*-PNR SPAD systems, the histograms $k_{l}$ approximately follows the multivariate normal distribution N(sNμ,sNC), so the log-likelihood function is

(A14) $$l\left(k_{l};\theta \right)=\ln P(k_{l},\theta )=-\frac{1}{2}\ln\left|sNC\right|-\frac{1}{2sN}\left(k_{l}-sN\mu \right)C^{-1}{\left(k_{l}-sN\mu \right)}^{T}$$

## References

[1] Liang D., Zhang C., Zhang P., Liu S., Li H., Niu S., Rao R.Z., Zhao L., Chen X., Li H. (2024). Evolution of Laser Technology for Automotive LiDAR, an Industrial Viewpoint. Nat. Commun..

[2] Kumagai O., Ohmachi J., Matsumura M., Yagi S., Tayu K., Amagawa K., Matsukawa T., Ozawa O., Hirono D., Shinozuka Y. 7.3 A 189 × 600 Back-Illuminated Stacked SPAD Direct Time-of-Flight Depth Sensor for Automotive LiDAR Systems. Proceedings of the 2021 IEEE International Solid-State Circuits Conference (ISSCC).

[3] Rapp J., Tachella J., Altmann Y., McLaughlin S., Goyal V.K. (2020). Advances in Single-Photon Lidar for Autonomous Vehicles: Working Principles, Challenges, and Recent Advances. IEEE Signal Process. Mag..

[4] Khan M.U., Zaidi S.A.A., Ishtiaq A., Bukhari S.U.R., Samer S., Farman A. A Comparative Survey of LiDAR-SLAM and LiDAR Based Sensor Technologies. Proceedings of the 2021 Mohammad Ali Jinnah University International Conference on Computing (MAJICC).

[5] Diab A., Kashef R., Shaker A. (2022). Deep Learning for LiDAR Point Cloud Classification in Remote Sensing. Sensors.

[6] Wang Z., Menenti M. (2021). Challenges and Opportunities in Lidar Remote Sensing. Front. Remote Sens..

[7] Villa F., Severini F., Madonini F., Zappa F. (2021). SPADs and SiPMs Arrays for Long-Range High-Speed Light Detection and Ranging (LiDAR). Sensors.

[8] Ma J., Zhuo S., Qiu L., Gao Y., Wu Y., Zhong M., Bai R., Sun M., Chiang P.Y. (2024). A Review of ToF-based LiDAR. J. Semicond..

[9] Wang Z., Yang X., Tian N., Liu M., Cai Z., Feng P., Dou R., Yu S., Wu N., Liu J. (2024). A 64 × 128 3D-Stacked SPAD Image Sensor for Low-Light Imaging. Sensors.

[10] Morimoto K., Iwata J., Shinohara M., Sekine H., Abdelghafar A., Tsuchiya H., Kuroda Y., Tojima K., Endo W., Maehashi Y. 3.2 Megapixel 3D-Stacked Charge Focusing SPAD for Low-Light Imaging and Depth Sensing. Proceedings of the 2021 IEEE International Electron Devices Meeting (IEDM).

[11] Zappa F., Tosi A., Mora A.D., Tisa S. (2009). SPICE Modeling of Single Photon Avalanche Diodes. Sens. Actuators A Phys..

[12] Zhang C., Zhang N., Ma Z., Wang L., Qin Y., Jia J., Zang K. (2022). A 240 × 160 3D-Stacked SPAD dToF Image Sensor with Rolling Shutter and In-Pixel Histogram for Mobile Devices. IEEE Open J. Solid-State Circuits Soc..

[13] Padmanabhan P., Zhang C., Cazzaniga M., Efe B., Ximenes A.R., Lee M.J., Charbon E. 7.4 A 256 × 128 3D-Stacked (45 nm) SPAD FLASH LiDAR with 7-Level Coincidence Detection and Progressive Gating for 100m Range and 10klux Background Light. Proceedings of the 2021 IEEE International Solid-State Circuits Conference (ISSCC).

[14] Hutchings S.W., Johnston N., Gyongy I., Al Abbas T., Dutton N.A.W., Tyler M., Chan S., Leach J., Henderson R.K. (2019). A Reconfigurable 3-D-Stacked SPAD Imager with In-Pixel Histogramming for Flash LIDAR or High-Speed Time-of-Flight Imaging. IEEE J. Solid-State Circuits.

[15] Incoronato A., Locatelli M., Zappa F. (2021). Statistical Modelling of SPADs for Time-of-Flight LiDAR. Sensors.

[16] Xia H., Yu X., Zhang J., Cao G. (2024). A Review of Advancements and Trends in Time-to-Digital Converters Based on FPGA. IEEE Trans. Instrum. Meas..

[17] Wang X., Zhou Z., Li C., Hu J., Li D., Ma R., Liu Y., Zhu Z. A 7.9 Ps Resolution, Multi-Event TDC Using an Ultra-Low Static Phase Error DLL and High Linearity Time Amplifier for dToF Sensors. Proceedings of the 2024 IEEE Custom Integrated Circuits Conference (CICC).

[18] Gyongy I., Hutchings S.W., Halimi A., Tyler M., Chan S., Zhu F., McLaughlin S., Henderson R.K., Leach J. (2020). High-Speed 3D Sensing via Hybrid-Mode Imaging and Guided Upsampling. Optica.

[19] Koerner L.J. (2021). Models of Direct Time-of-Flight Sensor Precision That Enable Optimal Design and Dynamic Configuration. IEEE Trans. Instrum. Meas..

[20] Scholes S., Mora-Martín G., Zhu F., Gyongy I., Soan P., Leach J. (2023). Fundamental Limits to Depth Imaging with Single-Photon Detector Array Sensors. Sci. Rep..

[21] Scholes S., Wade E., McCarthy A., Garcia-Armenta J., Tobin R., Soan P.J., Buller G.S., Leach J. (2024). Robust Framework for Modelling Long Range dToF SPAD Lidar Performance. Opt. Express.

[22] Coates P.B. (1968). The Correction for Photon `pile-up’ in the Measurement of Radiative Lifetimes. J. Phys. E Sci. Instrum..

[23] Rapp J., Ma Y., Dawson R.M.A., Goyal V.K. (2019). Dead Time Compensation for High-Flux Ranging. IEEE Trans. Signal Process..

[24] Choi Y.H., Kuc T.Y. Walk Error Compensation of ToF LiDAR Using Zero Crossing Discriminator with Auto Gain Control Amplifier. Proceedings of the 2022 22nd International Conference on Control, Automation and Systems (ICCAS).

[25] Huang K., Li S., Ma Y., Tian X., Zhou H., Zhang Z.-Y. (2018). Theoretical model and correction method of range walk error for single-photon laser ranging. Acta Phys. Sin..

[26] Yang R., Tang Y., Fu Z., Qiu J., Liu K. (2022). A Method of Range Walk Error Correction in SiPM LiDAR with Photon Threshold Detection. Photonics.

[27] Padmanabhan P., Zhang C., Charbon E. (2019). Modeling and Analysis of a Direct Time-of-Flight Sensor Architecture for LiDAR Applications. Sensors.

[28] Rapp J., Ma Y., Dawson R.M.A., Goyal V.K. (2021). High-Flux Single-Photon Lidar. Optica.

[29] Rech I., Ingargiola A., Spinelli R., Labanca I., Marangoni S., Ghioni M., Cova S. (2008). Optical Crosstalk in Single Photon Avalanche Diode Arrays: A New Complete Model. Opt. Express.

[30] Wojtkiewicz M., Rae B., Henderson R.K. (2024). Review of Back-Side Illuminated 3-D-Stacked SPADs for Time-of-Flight and Single-Photon Imaging. IEEE Trans. Electron Devices.

[31] Williams G.M. (2017). Optimization of Eyesafe Avalanche Photodiode Lidar for Automobile Safety and Autonomous Navigation Systems. Opt. Eng..

[32] (2023). Assessment Methodology for Automotive LiDAR Sensors.

[33] (2025). Automotive Lidar Performance Requirements and Test Methods.

[34] Cassanelli D., Cattini S., Loro G.D., Cecilia L.D., Ferrari L., Rovati L. LiDARs Detected Signal and Target Distance Estimation: Measurement Errors from Target Reflectance and Multiple Echos. Proceedings of the 2022 IEEE International Workshop on Metrology for Automotive (MetroAutomotive).

[35] Colomb M., Duthon P., Bernardin F. Spectral Reflectance Characterization of the Road Environment to Optimize the Choice of Autonomous Vehicle Sensors. Proceedings of the 2019 IEEE Intelligent Transportation Systems Conference (ITSC).

[36] Kashani A.G., Olsen M.J., Parrish C.E., Wilson N. (2015). A Review of LIDAR Radiometric Processing: From Ad Hoc Intensity Correction to Rigorous Radiometric Calibration. Sensors.

[37] Kay S. (1993). Fundamentals of Statistical Signal Processing, Volume I: Estimation Theory.

[38] Barndorff-Nielsen O.E., Cox D.R. (1989). Asymptotic Techniques for Use in Statistics.

[39] (2022).

[40] Liu Y., Zhao Y., Hu J., Ma R., Zhu Z. (2024). An Efficient and Comprehensive Timing Jitter Model for Single Photon Avalanche Diodes. IEEE Trans. Electron Devices.

[41] Xu L., Chang Y., Liu L., Qiao K., Xu Z., Wang J., Su C., Liu T., Yin F., Wang X. (2025). An Efficient Simplified SPAD Timing Jitter Model in Verilog-A for Circuit Simulation. Electronics.

[42] Frolkovič P. (1990). Numerical Recipes: The Art of Scientific Computing. Acta Appl. Math..

