# Supplementary material for: Performance Bounds of Ranging Precision in SPAD-Based dToF LiDAR
Source: Sensors (Basel). 2025 Oct 6;25(19):6184. doi: 10.3390/s25196184 (PMC12526853; doi:10.3390/s25196184)
Supplement: Supplementary file 1 [file sensors-25-06184-s001.zip › SI.pdf]

Supplementary Information: Performance Bounds of Ranging Precision in SPAD-Based dToF LiDAR

Hao Wu [1,2,*], Yingyu Wang[1], Shiyi Sun[1], Lijie Zhao[1], Limin Tong[2], Linjie Shen[1] and Jiang Zhu[1†]

[1] Hikvision Research Institute, Hangzhou 310051, China;

[2] State Key Laboratory of Extreme Photonics and Instrumentation, College of Optical Science and Engineering, Zhejiang University, Hangzhou 310027, China;

S1 Effects for Unequal Dead Times of TDC and SPAD

In the main text, for convenience in establishing the probabilistic model of the histogram generated in a dToF system, we assumed that the dead times of the TDC (T_e) and the SPAD (T_s) are equal. Here, we extend the discussion to cases where the two dead times differ.

First, consider the case where $T_\mathrm{e} < T_\mathrm{s}$, i.e., the TDC dead time is shorter than the SPAD dead time. Since the TDC is ready to be re-triggered while the SPAD is still inactive, the TDC cannot actually receive any new trigger until the SPAD recovers. Thus, the effective system dead time is still governed by the SPAD dead time. In other words, this case reduces to the situation described in the main text with $T = T_\mathrm{e,eff} = T_\mathrm{s}$.

Secondly, when $T_\mathrm{e} > T_\mathrm{s}$, i.e., the TDC dead time is longer than the SPAD dead time, the trigger behavior becomes more complicated. As shown in Fig. S1, a SPAD trigger also triggers the TDC, and both enter their respective dead times. Since $T_\mathrm{s} < T_\mathrm{e}$, the SPAD recovers earlier and becomes ready to fire again while the TDC is still inactive. If the SPAD fires again during this interval (e.g., at point B in the figure), the TDC cannot record this event; as such, it does not appear in the final histogram. However, this secondary SPAD trigger forces the SPAD back into dead time. By the time the TDC recovers, the SPAD may still be in its secondary dead time, leaving the system unable to trigger. The system can only resume after this additional SPAD dead time has passed.

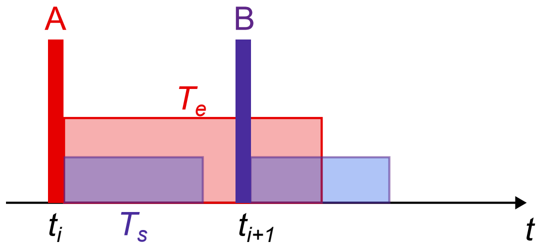

Figure S1: Illustration of the effect when $T_\mathrm{s} < T_\mathrm{e}$, where secondary SPAD triggering occurs at t_{i+1} (event B) within the TDC dead time induced by event A at t_i. As a result, even after the TDC dead time ends, the system remains in the SPAD secondary-trigger-induced dead time state.

Intuitively, this behavior introduces a hidden variable (whether and when the SPAD fires again during TDC dead time), which extends the effective dead time. Instead of being fixed at T_e, the

*wuhao_zju@zju.edu.cn
†zhujiang@hikvision.com

effective dead time can vary anywhere between T_e (if no secondary SPAD firing occurs) and $T_\mathrm{e}+T_\mathrm{s}$ (if a secondary firing occurs just before TDC recovery).

Following the approach in the main text, we now build a detection model for this case and use it to calculate the Fisher information and the CRLB. For simplicity, we restrict ourselves to the case $T_\mathrm{s} < T_\mathrm{e} \leq 2T_\mathrm{s}$, meaning that during the TDC dead time, the SPAD can fire at most once more. Larger T_e would allow multiple secondary SPAD triggers within a single TDC dead time, making the analysis more complex; however, such cases can be addressed with similar methods.

Unlike Eq. (3) in the main text, here, for the i-th bin, we must account for not only the reduction in available measurements due to TDC triggers within the past T_e bins, but also for the case where a TDC trigger in the past $T_\mathrm{e}+T_\mathrm{s}$ bins leads to a secondary SPAD trigger, leaving the i-th bin still in dead time. Therefore, for the i-th bin we have

$$P(h_i|h_{i-1}, h_{i-2}, ..., h_{i-T_\mathrm{e}-T_\mathrm{s}}) \sim \mathcal{B}(N', q_i), \tag{S1}$$

where N' represents the number of effective measurements available in the i-th bin. Unlike in the main text, N' here is no longer a constant but a random variable depending on the hidden variables:

$$N' = N - \sum_{j=1}^{T_\mathrm{e}} h_{i-j} - \sum_{j\in\mathcal{J}_i} f_{j,i}. \tag{S2}$$

Here, $\sum_{j=1}^{T_\mathrm{e}} h_{i-j}$ is the same as in the main text, accounting for the loss due to TDC dead time. The third term, $f_{j,i}$, denotes the additional loss of availability in bin i caused by a secondary SPAD trigger following a TDC trigger in bin j. The index set of such prior triggers is $\mathcal{J}_i = \{j | j \in [i-T_\mathrm{e}-T_\mathrm{s}, i-T_\mathrm{e}-1]\}$. Each $f_{j,i}$ follows a binomial distribution:

$$f_{j,i} \sim B\left(h_j, \sum_{k=i-T_\mathrm{e}-j}^{T_\mathrm{s}} G_{j,k}\right), \tag{S3}$$

where $G_{j,k}$ is the probability that after a TDC trigger in bin j, the bin $j+T_\mathrm{e}+k$ is the first bin unaffected by SPAD dead time, with a value of

$$G_{j,k} = \begin{cases} \prod_{r=j+T_\mathrm{s}+1}^{j+T_\mathrm{e}} p_r & k=0 \\ q_{j+T_\mathrm{e}+k-T_\mathrm{s}} \prod_{r=j+T_\mathrm{s}+1}^{j+T_\mathrm{e}+k-T_\mathrm{s}-1} p_r & k>0 \end{cases} \tag{S4}$$

We denote

$$g_{j,i} = \sum_{k=i-T_\mathrm{e}-j}^{T_\mathrm{s}} G_{j,k}, \tag{S5}$$

which represents the probability that bin i is still affected by SPAD dead time given a trigger at bin j.

Considering these expectations, it can be obtained that

$$E[h(i)] = E[N']q_i = q_i\left(N - \sum_{j=1}^{T_\mathrm{e}} E[h(i-j)] - \sum_{j=i-T_\mathrm{e}-1}^{i-T_\mathrm{e}-T_\mathrm{s}} E[f(j,i)]\right), \tag{S6}$$

with

$$E[f(j,i)] = E[h(j)]g_{j,i}. \tag{S7}$$

By analogy with the definition of F_i in the main text, we define $E[h(i)] = NQ_i = NF_iq_i$, giving

$$F_i = 1 - \sum_{j=1}^{T_\mathrm{e}} Q_{i-j} - \sum_{j=i-T_\mathrm{e}-1}^{i-T_\mathrm{e}-T_\mathrm{s}} Q_j g_{j,i}. \tag{S8}$$

In the steady-state scenario for $i<0$, it can be readily shown that

$$F_b = \frac{1}{1+p_b\left(T_\mathrm{e} + \sum_{k=1}^{T_\mathrm{s}} kG_{b,k}\right)} \tag{S9}$$

To validate the accuracy of our detection probability model, Fig. S2 compares histograms generated via Monte Carlo (MC) simulations with the corresponding NQ_i calculated from the model under typical parameters for the case $T_\mathrm{s} < T_\mathrm{e}$. As shown in the figure, the simulated histograms closely agree with the model's predictions, confirming the correctness of the detection probability model. For comparison, when contrasted with the theoretical histograms for $T_\mathrm{s} = T_\mathrm{e}$, two observations can be made: first, under steady-state conditions, the histogram counts for $T_\mathrm{s} < T_\mathrm{e}$ are lower than those for $T_\mathrm{s} = T_\mathrm{e}$; second, after the object peak, the counts recover more slowly when $T_\mathrm{s} < T_\mathrm{e}$. Both effects arise from the reduced number of available detections due to SPAD secondary triggering.

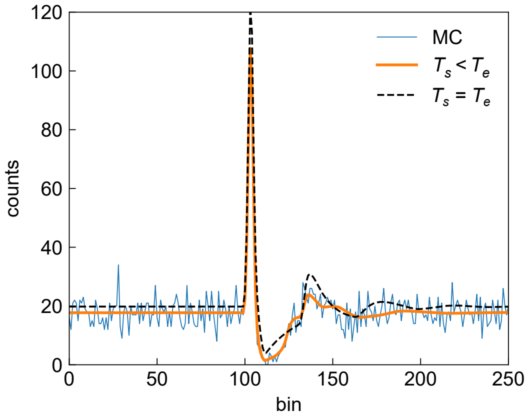

Figure S2: Comparison of histograms generated by Monte Carlo simulations (blue solid) and the detection probability model (orange solid) with $t_0 = 100\tau$, $w = 4\tau$, $R = 1/\tau$, $R_n = 0.05/\tau$, $T_\mathrm{s} = 20$, and $T_\mathrm{e} = 30$. For comparison, the histogram generated by the detection probability model with $T_\mathrm{e} = T_\mathrm{s} = 30$ is shown as a black dashed line.

We then proceed analogously to the main text. Because of the hidden variables, the complete conditional probability distribution of h_l becomes the marginal probability

$$H_l(h_l|h_{<l}) = \sum_{\boldsymbol{f}} P\{h_l|h_{<l}, \boldsymbol{f}\} P\{\boldsymbol{f}|h_{<l}\}, \tag{S10}$$

where $h_{<l}$ denotes the counts before l-th bin and $\boldsymbol{f} = [f_{j,l} \mid j \in \mathcal{J}_l]$ represents the set of hidden variables.

$$P\{h_l|h_{<l}, \boldsymbol{f}\} = B(h_l; N'_l, q_l) = \binom{N'_l}{h_l} q_l^{h_l} p_l^{N'_l - h_l}, \tag{S11}$$

denotes that h_l follows a binomial distribution conditioned on $\boldsymbol{f}$, and

$$P\{\boldsymbol{f}|h_{<l}\} = \prod_{j \in \mathcal{J}_l} B(f_{jl}; h_j, g_{j,l}), \tag{S12}$$

denotes that $\boldsymbol{f}$ also follows a binomial distribution conditioned on h_j and $g_{j,l}$.

Following the derivation in Eq. (19) of the main text, the derivative of H_l is taken, yielding

$$\begin{aligned} \frac{\partial H_l}{\partial \theta} &= \frac{\partial}{\partial \theta} \sum_{\boldsymbol{f}} P\{h_l|h_{<l}, \boldsymbol{f}; \theta\} P\{\boldsymbol{f}|h_{<l}; \theta\} \\ &= \sum_{\boldsymbol{f}} P\{h_l|h_{<l}, \boldsymbol{f}; \theta\} \frac{\partial}{\partial \theta} P\{\boldsymbol{f}|h_{<l}; \theta\} + P\{\boldsymbol{f}|h_{<l}; \theta\} \frac{\partial}{\partial \theta} P\{h_l|h_{<l}, \boldsymbol{f}; \theta\}. \end{aligned} \tag{S13}$$

The derivative of the binomial probability conditioned on $\boldsymbol{f}$ is analogous to Eq. (18) in the main text, and can be expressed as

$$\frac{\partial}{\partial \theta} P\{h_l|h_{<l}, \boldsymbol{f}; \theta\} = P\{h_l|h_{<l}, \boldsymbol{f}; \theta\} \frac{h_l - N'_l(\boldsymbol{f}) q_l}{q_l p_l} \frac{\partial q_l}{\partial \theta}. \tag{S14}$$

For the derivative with respect to the hidden variables, it can be expressed as

$$\frac{\partial}{\partial\theta}P\{\boldsymbol{f}|h_{<l};\theta\} = P\{\boldsymbol{f}|h_{<l};\theta\}\sum_{j\in\mathcal{J}_l}\frac{f_{j,l}-h_jg_{j,l}}{g_{j,l}(1-g_{j,l})}\frac{\partial g_{j,l}}{\partial\theta}. \tag{S15}$$

Substituting the above two expressions into the original formulation, it yields

$$\frac{\partial H_l}{\partial\theta} = \sum_{\boldsymbol{f}}P\{h_l|h_{<l},\boldsymbol{f};\theta\}P\{\boldsymbol{f}|h_{<l};\theta\}\left[\frac{h_l-N_l'(\boldsymbol{f})q_l}{q_lp_l}\frac{\partial q_l}{\partial\theta}+\sum_{j\in\mathcal{J}_l}\frac{f_{j,l}-h_jg_{j,l}}{g_{j,l}(1-g_{j,l})}\frac{\partial g_{j,l}}{\partial\theta}\right]. \tag{S16}$$

Accordingly, for the first term in Eq. (17) of the main text, we first denote

$$\sum_{\boldsymbol{h}_l}\frac{1}{H_l}\frac{\partial H_l}{\partial\theta_\alpha}\frac{\partial H_l}{\partial\theta_\beta} = E_{\boldsymbol{h}}\left[\frac{\partial\ln H_l}{\partial\theta_\alpha}\frac{\partial\ln H_l}{\partial\theta_\beta}\right], \tag{S17}$$

and

$$\begin{aligned}\frac{\partial\ln H_l}{\partial\theta} &= \frac{1}{H_l}\frac{\partial H_l}{\partial\theta}\\ &= \sum_{\boldsymbol{f}}\frac{P\{h_l|h_{<l},\boldsymbol{f};\theta\}P\{\boldsymbol{f}|h_{<l};\theta\}}{H_l}\left[\frac{h_l-N_l'(\boldsymbol{f})q_l}{q_lp_l}\frac{\partial q_l}{\partial\theta}+\sum_{j\in\mathcal{J}_l}\frac{f_{j,l}-h_jg_{j,l}}{g_{j,l}(1-g_{j,l})}\frac{\partial g_{j,l}}{\partial\theta}\right]\\ &= \sum_{\boldsymbol{f}}w(\boldsymbol{f}|h_l,h_{<l})\left[\frac{h_l-N_l'(\boldsymbol{f})q_l}{q_lp_l}\frac{\partial q_l}{\partial\theta}+\sum_{j\in\mathcal{J}_l}\frac{f_{j,l}-h_jg_{j,l}}{g_{j,l}(1-g_{j,l})}\frac{\partial g_{j,l}}{\partial\theta}\right]\\ &= E_{\boldsymbol{f}\sim w|h_l,h_{<l}}\left[\frac{h_l-N_l'(\boldsymbol{f})q_l}{q_lp_l}\frac{\partial q_l}{\partial\theta}+\sum_{j\in\mathcal{J}_l}\frac{f_{j,l}-h_jg_{j,l}}{g_{j,l}(1-g_{j,l})}\frac{\partial g_{j,l}}{\partial\theta}\right],\end{aligned} \tag{S18}$$

where $E_{\boldsymbol{f}\sim w|h_l,h_{<l}}[\cdot]$ denotes the expectation with respect to the posterior distribution of $\boldsymbol{f}$, conditioned on h_l and $h_{<l}$.

For simplicity of analysis, let

$$\begin{aligned}s_1(h_i,\boldsymbol{f}) &= \frac{h_l-N_l'(\boldsymbol{f})q_l}{q_lp_l}\frac{\partial q_l}{\partial\theta}\\ s_2(\boldsymbol{f}) &= \sum_{j\in\mathcal{J}_l}\frac{f_{j,l}-h_jg_{j,l}}{g_{j,l}(1-g_{j,l})}\frac{\partial g_{j,l}}{\partial\theta},\end{aligned} \tag{S19}$$

and the first term can then be written as

$$\frac{\partial\ln H_l}{\partial\theta} = E_{\boldsymbol{f}\sim w|h_l,h_{<l}}[s_1+s_2]. \tag{S20}$$

Therefore, for Eq. (19) in the main text, applying the law of total variance yields

$$E_{h_l,h_{<l}}[(E_{\boldsymbol{f}\sim w|h_l,h_{<l}}[s_1+s_2])^2] = E_{h_l,\boldsymbol{f},h_{<l}}[(s_1+s_2)^2] - E_{h_l,h_{<l}}[\mathrm{Var}_{\boldsymbol{f}\sim w|h_l,h_{<l}}(s_1+s_2)], \tag{S21}$$

where the first term represents the expectation over the prior distribution of $\boldsymbol{f}$, while the second term corresponds to the variance of $\boldsymbol{f}$ under its posterior distribution, conditioned on h_l.

Expanding the first term yields three components: s_1^2,s_1s_2 and s_2^2:

1) For $E_{h_l,\boldsymbol{f},h_{<l}}[s_1^2]$, similar to Eq. (A4) in the main text, we finally obtain

$$E_{h_l,\boldsymbol{f},h_{<l}}[s_1^2] = \frac{NF_l}{q_lp_l}\frac{\partial q_l}{\partial\theta_\alpha}\frac{\partial q_l}{\partial\theta_\beta}. \tag{S22}$$

2) For $E_{h_l,\boldsymbol{f},h_{<l}}[s_1s_2]$, we first condition on $\boldsymbol{f}$, yielding

$$E_{h_l,\boldsymbol{f},h_{<l}}[s_1s_2] = E_{h_{<l}}[E_{h_l,\boldsymbol{f}}[s_1s_2|\boldsymbol{f}]] = E_{h_{<l}}[s_2E_{h_l,\boldsymbol{f}}[s_1|\boldsymbol{f}]]. \tag{S23}$$

Since $E_{h_l,\boldsymbol{f}}[h_l|\boldsymbol{f}] = N_l(\boldsymbol{f})q_l$, it follows that $E_{h_l,\boldsymbol{f}}[s_1|\boldsymbol{f}] = 0$, which leads to $E_{h_l,\boldsymbol{f},h_{<l}}[s_1 s_2] = 0$.

3) For $E_{h_l,\boldsymbol{f},h_{<l}}[s_2^2]$, since s_2 depends only on $h_{<l}$ and $\boldsymbol{f}$, we have

$$
\begin{aligned}
E_{h_l,\boldsymbol{f},h_{<l}}[s_2^2] &= E_{h_{<l}}[E_{\boldsymbol{f}|h_{<l}}[s_2^2]] \\
&= E_{h_{<l}}\left[\sum_{j\in\mathcal{J}_l} \frac{E[(f_{j,l}-h_j g_{j,l})^2|h_{<l}]}{g_{j,l}^2(1-g_{j,l}^2)} \frac{\partial g_{j,l}}{\partial\theta_\alpha}\frac{\partial g_{j,l}}{\partial\theta_\beta}\right]
\end{aligned} \tag{S24}
$$

Given $h_{<l}$, $f_{j,l}|h_{<l} \sim B(h_j, g_{j,l})$, it can be immediately obtained that $E[(f_{j,l}-h_j g_{j,l})^2|h_{<l}] = h_j g_{j,l}(1-g_{j,l})$, and thus

$$
E_{h_l,\boldsymbol{f},h_{<l}}[s_2^2] = N\sum_{j\in\mathcal{J}_l} \frac{F_j q_j}{g_{j,l}(1-g_{j,l})}\frac{\partial g_{j,l}}{\partial\theta_\alpha}\frac{\partial g_{j,l}}{\partial\theta_\beta}. \tag{S25}
$$

For the posterior variance term $E_{h_l,h_{<l}}[\mathrm{Var}_{\boldsymbol{f}\sim w|h_l,h_{<l}}(s_1+s_2)]$, expressing s_1+s_2 as a linear function of $\boldsymbol{f}$, yields

$$
s = s_1 + s_2 = \text{const.} + \sum_{j\in\mathcal{J}_l} c_{j,l} f_{j,l}, \tag{S26}
$$

where

$$
c_{j,l} = \frac{1}{p_l}\frac{\partial q_l}{\partial\theta_\alpha} + \frac{1}{g_{j,l}(1-g_{j,l})}\frac{\partial g_{j,l}}{\partial\theta_\alpha}. \tag{S27}
$$

Therefore the posterior variance can be expressed as

$$
\mathrm{Var}_{\boldsymbol{f}\sim w|h_l,h_{<l}}(s_1+s_2) = \sum_{j,k\in\mathcal{J}_l} c_{j,l}c_{k,l}\,\mathrm{Cov}_{\boldsymbol{f}\sim w|h_l,h_{<l}}(f_{j,l}, f_{k,l}). \tag{S28}
$$

In the case of low ambient light and a large number of measurements (large-N approximation), the posterior distribution of $\boldsymbol{f}$ can be approximated by its prior distribution, i.e., $f_{j,l}|h_l, h_{<l} \approx f_{j,l}|h_{<l} \sim B(h_j, g_{j,l})$, which leads to

$$
\begin{aligned}
& E[\mathrm{Var}_{\boldsymbol{f}\sim w|h_l,h_{<l}}(s_1+s_2)] \\
\approx\ & \sum_{j\in\mathcal{J}_l} E[\mathrm{Var}_{\boldsymbol{f}\sim w|h_{<l}}(c_{j,l}f_{j,l})] \\
=\ & \sum_{j\in\mathcal{J}_l} E[c_{j,l}^2 h_j g_{j,l}(1-g_{j,l})] \\
=\ & N\sum_{j\in\mathcal{J}_l} F_j q_j \left[\frac{g_{j,l}(1-g_{j,l})}{p_l^2}\frac{\partial q_l}{\partial\theta_\alpha}\frac{\partial q_l}{\partial\theta_\beta} + \frac{1}{p_l}\left(\frac{\partial q_l}{\partial\theta_\alpha}\frac{\partial g_{j,l}}{\partial\theta_\beta} + \frac{\partial q_l}{\partial\theta_\beta}\frac{\partial g_{j,l}}{\partial\theta_\alpha}\right) + \frac{1}{g_{j,l}(1-g_{j,l})}\frac{\partial g_{j,l}}{\partial\theta_\alpha}\frac{\partial g_{j,l}}{\partial\theta_\beta}\right].
\end{aligned} \tag{S29}
$$

Noting that the last term in the above expression is identical to the corresponding term $E[s_2^2]$ under the prior distribution, it cancels out. Consequently, we obtain the final expression, which can be interpreted as the contribution of the l-th bin to the Fisher information, denoted by$S_{\alpha\beta}(l)$:

$$
\begin{aligned}
& S_{\alpha\beta}(l) \\
=\ & \sum_{\boldsymbol{h}_l} \frac{1}{H_l}\frac{\partial H_l}{\partial\theta_\alpha}\frac{\partial H_l}{\partial\theta_\beta} \\
\approx\ & \frac{NF_l}{q_l p_l}\frac{\partial q_l}{\partial\theta_\alpha}\frac{\partial q_l}{\partial\theta_\beta} - N\sum_{j\in\mathcal{J}_l} F_j q_j \left[\frac{g_{j,l}(1-g_{j,l})}{p_l^2}\frac{\partial q_l}{\partial\theta_\alpha}\frac{\partial q_l}{\partial\theta_\beta} + \frac{1}{p_l}\left(\frac{\partial q_l}{\partial\theta_\alpha}\frac{\partial g_{j,l}}{\partial\theta_\beta} + \frac{\partial q_l}{\partial\theta_\beta}\frac{\partial g_{j,l}}{\partial\theta_\alpha}\right)\right].
\end{aligned} \tag{S30}
$$

The final result can be divided into three terms.

1) The first term, proportional to $\frac{\partial q_l}{\partial\theta_\alpha}\frac{\partial q_l}{\partial\theta_\beta}$, represents the contribution of the binomial variance of the histogram count for a single bin, given $\boldsymbol{f}$. This term is consistent in form with Eq. (21) in the main text, except that here F_l is reduced due to the additional count loss from secondary SPAD triggers, making it smaller than the corresponding F_l in the main text.

2) The second term, proportional to $\frac{\partial g_{j,l}}{\partial\theta_\alpha}\frac{\partial g_{j,l}}{\partial\theta_\beta}$, captures the fluctuations of hidden variables induced by secondary triggers within the dead-time range of bins in $\mathcal{J}_l$, which reduces the contribution of the first term.

3) The third term, proportional to $\frac{\partial q_l}{\partial \theta_\alpha}\frac{\partial g_{j,l}}{\partial \theta_\beta}$, reflects the coupling of information when the parameter θ simultaneously affects both q_l and $g_{j,l}$.

It can be also noted that the second and third terms are of order $O(NQ_b(T_\mathrm{e}-T_\mathrm{s}))$, corresponding approximately to the probability of a secondary SPAD trigger occurring within the TDC dead time. Consequently, in the limit $R_n \to 0$ or when $T_\mathrm{e} = T_\mathrm{s}$, only the first term contributes to the Fisher information, which coincides with Eq. (21) in the main text; therefore, we can use the first term as an optimistic estimation of the Fisher information.

Figure S3 shows the variation in δt_0 with R_n for the cases $T_\mathrm{s} < T_\mathrm{e}$ and $T_\mathrm{s} = T_\mathrm{e}$, while keeping T_e constant, under typical parameters. It can be seen that δt_0 is consistently higher for $T_\mathrm{s} < T_\mathrm{e}$ than for $T_\mathrm{s} = T_\mathrm{e}$, indicating a degradation in ranging performance due to the presence of the latent variable associated with SPAD secondary triggering. Furthermore, as R_n increases, the probability of secondary triggering also increases, leading to greater performance degradation. The figure also presents the standard deviations of parameter estimates obtained via MC simulations combined with the estimation method. Here, however, due to the presence of latent variables $\boldsymbol{f}$, a maximum likelihood estimation (MLE) would theoretically require summing over all possible values of $\boldsymbol{f}$ (see Eq. S10), which entails a prohibitively large computational cost. Therefore, parameter estimation was performed using a least-squares approach, i.e.,

$$\hat{\boldsymbol{\theta}} = \underset{\boldsymbol{\theta}}{\operatorname{argmin}} \|N\boldsymbol{Q}(\boldsymbol{\theta}) - \boldsymbol{h}\|^2. \tag{S31}$$

Since the least-squares method cannot theoretically reach the CRLB, the estimated standard deviations exceed the theoretical CRLB values. Nevertheless, this provides indirect confirmation of the validity of the CRLB bounds presented in this work.

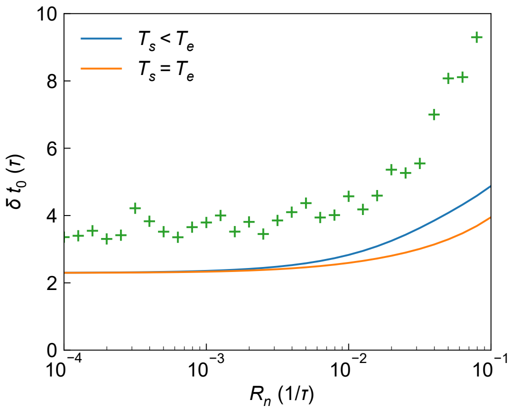

Figure S3: Theoretical ranging precision δt_0 with $T_s = 20$ (blue line) and $T_s = 30$ (orange line) as a function of the ambient photon flux rate R_n with laser FWHM of $w = 4\tau$, $t_0 = 100\tau$ and $T_e = 30$. The standard deviations of parameters estimated from histograms generated via MC simulations using the least-squares method are also shown for comparison (green crosses).

S2 Effects of Afterpulsing

Afterpulsing arises from carriers trapped in the SPAD, which may be released after a delay, generating correlated counts in subsequent time bins. Here, we extend our discussion in the main text to cases where afterpulsing is present.

We start with the following assumptions: 1) The afterpulsing probability is small, so cascaded afterpulsing events (i.e., afterpulses triggering further afterpulses) can be neglected [1, 2]. 2) The afterpulsing coefficient is determined solely by the system itself and is independent of the parameters $[\boldsymbol{\theta} = (t_0, R)]$ to be estimated. 3) Within a single bin interval, a SPAD detection event may arise from either a returning photon or an afterpulse; however, even if both occur simultaneously, the system records only one detection.

Suppose that a SPAD event occurs in bin j due to a laser pulse. Then, this event may generate an additional "photon-triggered" count in a subsequent $(j + r)$-th bin with probability $a(r)$. Following

the derivation in Sec. S1, the conditional probability for the i-th bin can be written as

$$H_i(h_i|h_{<i}) = \sum_{\boldsymbol{f}} P\{h_i|h_{<i}, \boldsymbol{f}\}P\{\boldsymbol{f}|h_{<i}\}, \tag{S32}$$

where $\boldsymbol{f} = \{f_{j,i}|j \in \mathcal{J}_i\}$ denotes the number of afterpulse counts in bin i originating from counts in bin j and $\mathcal{J}_i$ theoretically includes all bins prior to $i - T$, with $f_{j,i} \sim B(h_j, a(i-j))$. In practice, however, $a(r)$ is typically very small and decays exponentially with r, so only bins close to i contribute significantly to afterpulse counts.

Given the history $(h_{<i}, \boldsymbol{f})$, the counts in the i-th bin, h_i, can be attributed to two sources: 1) detection events triggered by afterpulses in the i-th bin, with total number $M_i = \sum_j f_{j,i}$; 2) detection events not associated with afterpulses but generated by received laser pulse, modeled as a random variable X. Accordingly, the count in the i-th bin can be expressed as

$$h_i = M_i + X_i \tag{S33}$$

Conditioned on $\boldsymbol{f}$, the random variable X follows a standard binomial distribution:

$$X_i \sim B(N_i' - M_i, q_i) \tag{S34}$$

where

$$N_i' = N - \sum_{m=1}^{T} h_{i-m} \tag{S35}$$

represents the number of available detection trials not blocked by dead time from the preceding T bins. Since afterpulse-triggered detections occupy trials regardless of whether a returning photon is also present, the available trials for X_i are further reduced by M_i. Combining these results, the conditional probability of h_i can be written as

$$P\{h_i|h_{<i}, \boldsymbol{f}\} \sim Pr(X_i = h_i - M_i) \sim B(N' - \sum_{j\in\mathcal{J}_i} f_{j,i}, q_i). \tag{S36}$$

For $P\{\boldsymbol{f}|h_{<i}\}$, it can be expressed approximately as

$$P\{\boldsymbol{f}|h_{<i}\} \sim \prod_{j\in\mathcal{J}_l} P\{f_{j,l}|h_{<i}\}. \tag{S37}$$

For the distribution of $f_{j,l}$, we approximate

$$f_{j,l} \sim B(h_j, g_{j,l}), \tag{S38}$$

where $g_{j,l}$ incorporates both the afterpulsing probability $a(l-j)$ and corrections due to the prior history $h_{<i}$ (since intervening triggers between the j-th and i-th bins may suppress the registration of the afterpulse). However, because the afterpulsing probability is typically small (e.g., $< 10^{-3}$), we approximate $g_{j,l} \approx a(l-j)$.

Letting $E[h(i)] = NQ_i$, we have

$$Q_i = \left(1 - \sum_{m=1}^{T} Q_{i-m} - \frac{E[M_i]}{N}\right) q_i + \frac{E[M_i]}{N} \tag{S39}$$

where $E[M_i] = N\sum_{m=1}^{a_m} Q_{i-T-m}c(T+a_m)$ and a_m denotes the data corresponding to the bins where $a(\cdot)$ is significant. Defining $F_i = 1 - \sum_{m=1}^{T} Q_{i-m} - \sum_{m=1}^{a_m} Q_{i-T-m}c(T+a_m)$ the reduced factor as in the main text, we note that now $Q_i > F_i q_i$ because Q_i also includes counts due to afterpulsing.

In the steady-state scenario for $i < 0$, it can be readily shown that

$$Q_b = \frac{q_b}{1 + q_b T - \alpha(1 - q_b)} \tag{S40}$$

where $\alpha = \sum a(i)$ is the total afterpulsing probability.

To validate the accuracy of our detection probability model, Fig. S4 compares histograms generated via MC simulations with the corresponding NQ_i calculated from the model under typical parameters. Here, it is assumed that afterpulses are significant only in the two bins immediately following the end of the dead time, with $a(T) = 0.05$ and $a(T+1) = 0.005$. The simulated histograms agree closely with the model predictions, confirming the correctness of the detection probability model. For comparison, when contrasted with the theoretical histograms without afterpulses ($a = 0$), two observations can be made: first, under steady-state conditions, the histogram counts with $a > 0$ are higher than those with $a = 0$; second, during the recovery phase following the dead time of the object peak, the counts remain higher for $a > 0$. Both effects arise from the increased counts due to afterpulsing.

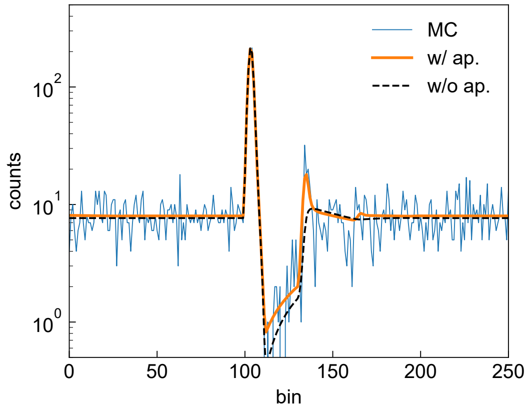

Figure S4: Comparison of histograms generated by MC simulations (blue solid) and the detection probability model (orange solid) with $t_0 = 100\tau$, $w = 4\tau$, $R = 1/\tau$, $a(T) = 0.05$, $a(T+1) = 0.005$ and $T = 30$. For comparison, the histogram generated by the detection probability model with $a = 0$ is shown as a black dashed line.

To compute the Fisher information matrix, we continue by evaluating $\partial H_i/\partial \boldsymbol{\theta}$. Analogous to the derivation in Section S1,

$$\frac{\partial H_l}{\partial \boldsymbol{\theta}} = \sum_{\boldsymbol{f}} w(\boldsymbol{f}|h_l, h_{<l}) \left[\frac{h_l - \sum_j f_{j,l} - N_l' q_l}{q_l p_l} \frac{\partial q_l}{\partial \boldsymbol{\theta}} + \sum_{j \in \mathcal{J}_l} \frac{f_{j,l} - h_j g_{j,l}}{g_{j,l}(1 - gj, l)} \frac{\partial g_{j,l}}{\partial \boldsymbol{\theta}} \right], \tag{S41}$$

where $g_{j,l} \approx a(l-j)$ denotes the afterpulse probability of bin j contributing to bin l. Since we assume $a(l-j)$ is independent of $\boldsymbol{\theta}$, only the first term remains:

$$\frac{\partial H_l}{\partial \boldsymbol{\theta}} = \sum_{\boldsymbol{f}} w(\boldsymbol{f}|h_l, h_{<l}) \left[\frac{h_l - \sum_j f_{j,l} - N_l' q_l}{q_l p_l} \frac{\partial q_l}{\partial \boldsymbol{\theta}} \right]. \tag{S42}$$

Let

$$s = \frac{h_l - M_l - (N_l' - M_l) q_l}{q_l p_l} \frac{\partial q_l}{\partial \boldsymbol{\theta}}, \tag{S43}$$

then, analogous to Eq. (19) in the main text,

$$E_{h_l, h_{<l}}[(E_{\boldsymbol{f} \sim w|h_l, h_{<l}}[s])^2] = E_{h_l, \boldsymbol{f}, h_{<l}}[s^2] - E_{h_l, h_{<l}}[\mathrm{Var}_{\boldsymbol{f} \sim w|h_l, h_{<l}}(s)], \tag{S44}$$

For the first term, given $\boldsymbol{f}$,

$$E_{h_l|\boldsymbol{f}, h_{<l}}[(h_l - M_l - (N_l' - M_l) q_l)^2] = \mathrm{Var}(X_l|\boldsymbol{f}) = (N_l' - M_l) q_l p_l. \tag{S45}$$

Taking expectation over $\boldsymbol{f}$ and $h_{<l}$,

$$E_{h_l, \boldsymbol{f}, h_{<l}}[(h_l - M_l - (N_l' - M_l) q_l)^2] = E[(N_l' - M_l) q_l p_l] = N F_l q_l p_l, \tag{S46}$$

so that

$$E_{h_l, \boldsymbol{f}, h_{<l}}[s^2] = \frac{N F_l}{q_l p_l} \frac{\partial q_l}{\partial \theta_\alpha} \frac{\partial q_l}{\partial \theta_\beta}. \tag{S47}$$

For the second term, the posterior variance, s, can be expressed linearly in $f_{j,l}$:

$$s = \text{const.} + \sum_{j\in\mathcal{J}_l} c_j f_{j,l}, \quad c_j = \frac{1}{q_l}\frac{\partial q_l}{\partial \boldsymbol{\theta}}. \tag{S48}$$

Hence,

$$E_{h_l,h_{<l}}[\mathrm{Var}_{\boldsymbol{f}\sim w|h_l,h_{<l}}(s)] = \sum_{j,k\in\mathcal{J}_l} c_j c_k E_{h_l,h_{<l}}[\mathrm{Cov}_{\boldsymbol{f}\sim w|h_l,h_{<l}}(f_{j,l}, f_{k,l})] \tag{S49}$$

Since $f_{j,l} \approx h_j a(l-j)$, the diagonal terms of $\mathrm{Cov}(f_{j,l}, f_{k,l})$ can be approximated as $\mathrm{Var}(f_{j,l}) \sim O(NQ_b\alpha(1-\alpha))$, while the off-diagonal terms can be approximated as $\mathrm{Cov}(f_{j,l}, f_{k,l}) \sim O(NQ_b^2\alpha^2)$. Here, Q_b denotes the detection probability per bin in the presence of background light only. Therefore, we neglect the off-diagonal terms and focus exclusively on the diagonal terms.

Overall, the Fisher information contributed by the l-th bin in the presence of afterpulsing can be approximated as

$$S_{\alpha\beta}(l) = \frac{NF_l}{q_l p_l}\frac{\partial q_l}{\partial \theta_\alpha}\frac{\partial q_l}{\partial \theta_\beta} - NO(Q_b\alpha). \tag{S50}$$

The first term is identical to the corresponding term in the main text, except that the effective number of available counts F_l now accounts for the reduction due to afterpulsing. The second term, $NO(Q_b\alpha)$, represents the information loss caused by the uncertainty due to afterpulses. Although the second term is difficult to evaluate, it is always negative. Therefore, we consider only the first term as an optimistic estimate of the Fisher information.

Figure S5 shows the variation in δt_0 with $a(T)$ under typical parameters. As in Fig. S4, it is assumed that afterpulses are significant only in the two bins immediately following the end of the dead time, with $a(T+1) = a(T)/10$. It can be seen that, as $a(T)$ increases, the standard deviation of the t_0 estimate increases, indicating a degradation in ranging performance. However, since the afterpulse probability remains very small, the performance deterioration is limited. Similar to the previous section, the figure also presents the standard deviations obtained from MC simulations combined with the least-squares method. Once again, because the least-squares method cannot theoretically reach the CRLB, the estimated standard deviations exceed the theoretical CRLB values, which indirectly confirms the validity of the CRLB bounds provided in this work.

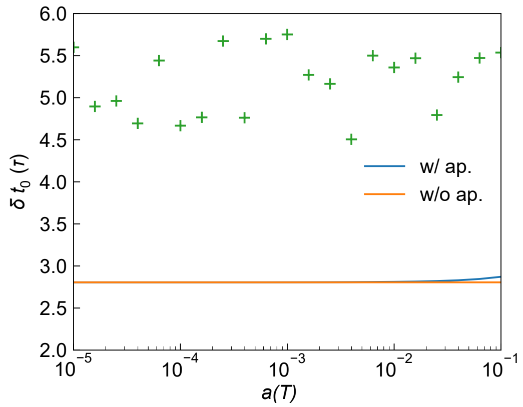

Figure S5: Theoretical ranging precision δt_0 with $a > 0$ (blue line) and $a = 0$ (orange line) as a function of the $a(T)$ with $a(T+1) = a(T)/10$, laser FWHM of $w = 4\tau$, $t_0 = 100\tau$ and $T = 30$. The standard deviations of parameters estimated from histograms generated via MC simulations using the least-squares method are also shown for comparison (green crosses).

S3 Effects for Crosstalk

When the detector is not a single SPAD pixel but an array composed of multiple SPAD pixels, optical crosstalk occurs when photons detected by one pixel actually originate from the optical paths associated with its neighboring pixels [3]. In the context of time-of-flight ranging, this phenomenon can be

interpreted as a modification of the observed received waveform of laser pulse. The measured waveform of signal is effectively the superposition of the true waveform of the pixel of interest and a scaled copy of the waveform observed by adjacent pixels. Depending on the distribution of distances within the field of view associated with different pixels, the superimposed waveform may exhibit spurious peaks that correspond to “false objects” not observable by the pixel itself. Alternatively, variations in the propagation lengths of the crosstalk paths may introduce relative delays to the peaks originating from the same object, which results in a broadened laser return waveform and consequently increases the uncertainty in the ranging result. In other words, the information content per detected photon is not fundamentally reduced, but the estimator is effectively provided with a distorted signal.

S4 Effects of Timing Jitter

Timing jitter in dToF systems refers to the uncertainty in the emitting time of the laser pulse due to the temporal reponse of the driver or the recorded photon arrival time due to the detector's temporal response [4]. In the context of Fisher information, timing jitter can be approximately modeled as an additional independent source of variance in the estimated parameter, especially for the time-of-flight t_0. Assuming the jitter is independent of other noise sources, its effect can be incorporated as an additive variance term. Denoting the variance due to timing jitter as σ^2_{jitter}, the total variance for the estimated parameter t_0 can be approximated as

$$\text{Var}(\hat{t}_0)_{\text{total}} \approx \text{Var}(\hat{t}_0)_{\text{CRLB}} + \frac{\sigma^2_{\text{jitter}}}{N} = \frac{(\delta t_0)^2 + \sigma^2_{\text{jitter}}}{N}, \tag{S51}$$

where $\text{Var}(t_0)_{\text{CRLB}}$ is the ideal CRLB computed from the Fisher information without timing jitter.

S5 Asynchronous Triggering of s-PNR Detectors

For s-PNR SPAD detectors, the individual subpixels are generally independent, meaning that under photon incidence, each subpixel can trigger asynchronously. As shown in Fig. S6, compared with synchronous triggering, asynchronous triggering can lead to situations where, at a given TDC-triggerable moment, some subpixels are still in the dead time caused by previous asynchronous triggers. If a photon arrives at this moment, the number of ”effectively available subpixels”, s_{eff}, is less than s, and s_{eff} decreases as the background noise intensity R_n increases.

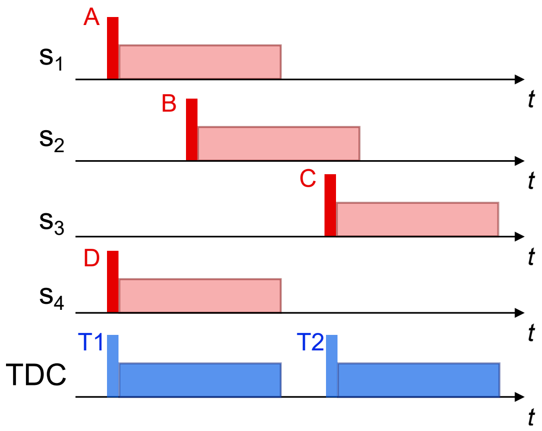

Figure S6: Effective number of triggerable subpixels, s_{eff}, reduced by asynchronous triggering in an s-PNR detector. Here, s_1-s_4 are four subpixels; s_1 and s_4 trigger simultaneously to produce TDC event $T1$. During the TDC dead time, s_2 triggers asynchronously (event B) but is not recorded. After the dead time, only s_1, s_3, and s_4 are triggerable, giving $s_{\text{eff}} = 3$, which reduces the probability of the next TDC event $T2$.

When $R_n \to 0$, before the arrival of the laser pulse echo (i.e., for $t < t_0$), the SPAD and TDC are almost never triggered, so $s_{\text{eff}} = s$. Therefore, during the duration of the return pulse, all subpixels

are in a triggerable state. In this regime, asynchronous triggering can be approximated as synchronous triggering, allowing the detection probability model and the resulting CRLB to be obtained using the derivations presented in the main text.

When the background noise intensity is non-negligible, the detection probability model becomes significantly more complex. Here, a Type I system is considered and analyzed qualitatively. Following the approach in Sec. S1, let the latent variable $\boldsymbol{f}$ denote the unrecorded asynchronous triggers occurring in previous bins within the TDC dead time. The system detection probability can then be expressed as

$$K_l(k_l|k_{<l}, h_{\leq l}) = \sum_{\boldsymbol{f}} P\{k_l|h_{\leq l}, \boldsymbol{f}\} P\{\boldsymbol{f}|k_{<l}, h_{<l}\}, \tag{S52}$$

where $P\{k_l|h_{\leq l}, \boldsymbol{f}\}$ is the probability of observing k_l counts in the current bin given the latent variables and historical TDC triggers. It follows a binomial distribution:

$$P\{k_l|h_{\leq l}, \boldsymbol{f}\} \sim B(s_{\text{eff}} N_l', u_l). \tag{S53}$$

where s_{eff} is a function of $\boldsymbol{f}$, reflecting the reduction in available subpixels in bin l due to asynchronous triggering in the range of bins $[l - T, l - 2T]$. The term $P\{\boldsymbol{f} \mid k_{<l}, h_{<l}\}$ represents the posterior distribution of $\boldsymbol{f}$ given the historical triggers.

Following the derivation in Sec. S1, the leading term of the system Fisher information can be written as

$$S^{(0)}_{\alpha\beta}(l) = s_{\text{eff}} N \frac{F_l}{u_l v_l} \frac{\partial u_l}{\partial \theta_\alpha} \frac{\partial u_l}{\partial \theta_\beta}, \tag{S54}$$

while the correction term should subtract the reduction in information caused by the uncertainty in s_{eff}.

Since $s_{\text{eff}} \leq s$, the Fisher information obtained for synchronous triggering in the main text [Eq. (30)] can be regarded as an optimistic estimate for the asynchronous case. Accordingly, the CRLB derived from this synchronous-trigger assumption provides a relatively loose lower bound for systems with asynchronous triggering.

S6 Error Analysis under Large-N Approximation in Type II Systems

In the main text, the Fisher information for Type II systems was calculated using the so-called large-N limit approximation. Specifically, in the limit of an infinitely large number of measurements N, the system detection probability model is assumed to follow a multivariate normal distribution, $\mathcal{N}(sN\boldsymbol{Q}, sN\boldsymbol{C})$, where $\boldsymbol{Q}$ is the vector of expected values and $\boldsymbol{C}$ is the covariance matrix; both $\boldsymbol{Q}$ and $\boldsymbol{C}$ are functions of the parameters $\boldsymbol{\theta}$ to be estimated. This approach leads to the Fisher information expression presented in Eq. (34) of the main text. Here, we discuss the order of errors introduced by this approximation. To simplify the analysis, a scalar case is considered.

Consider the standardized random variable

$$\boldsymbol{z} = \frac{1}{\sqrt{sN}} \left(k - sNQ(\theta) \right). \tag{S55}$$

According to the Edgeworth expansion, we have [5]

$$p_Z(z;\theta) = \varphi_C(z) \left[1 + \frac{1}{\sqrt{sN}} P_1(z;\theta) + O\left(\frac{1}{sN} \right) \right], \tag{S56}$$

where $\varphi_C(z)$ denotes a Gaussian distribution with covariance $sN\boldsymbol{C}$, and $P_1(z;\theta)$ is given by

$$P_1(z) = \frac{1}{6} \kappa H_3(z), \tag{S57}$$

where κ denotes the skewness (third-order cumulant) of the system distribution, and H_3 is the third-order Hermite polynomial. Returning to the distribution in terms of k, $p_k(k;\theta)$ does not change the order of N in the terms inside $[\cdot]$.

The log-likelihood for $p_k(k;\theta)$ is then given by

$$l_k(\theta) = \ln p_k(k;\theta) = \ln \varphi_{sNQ,sNC}(k;\theta) + \ln\left(1 + \frac{1}{\sqrt{sN}}P_1 + \cdots\right). \tag{S58}$$

let $l_k^{(G)} = \ln \varphi_{sNQ,sNC}(k;\theta)$, and according to $\ln(1+u) \approx u$,

$$l_k(\theta) \approx l_k^{(G)} + \frac{1}{\sqrt{sN}}P_1. \tag{S59}$$

Taking the derivative with respect to θ yields the score function

$$\frac{\partial l_k}{\partial\theta} \approx \frac{\partial l_k^{(G)}}{\partial\theta} + \frac{1}{\sqrt{sN}}\frac{\partial P_1}{\partial\theta}. \tag{S60}$$

And so the Fisher information can be expressed as

$$\begin{aligned} I(\theta) &= E\left[\left(\frac{\partial l_k}{\partial\theta}\right)^2\right] \\ &\approx E\left[\left(\frac{\partial l_k^{(G)}}{\partial\theta}\right)^2\right] + \frac{2}{\sqrt{sN}}E\left[\frac{\partial l_k^{(G)}}{\partial\theta}\frac{\partial P_1}{\partial\theta}\right] + \frac{1}{sN}E\left[\left(\frac{\partial P_1}{\partial\theta}\right)^2\right], \end{aligned} \tag{S61}$$

where the first term corresponds to the Fisher information under large-N approximation (i.e., Eq. (34) in the main text), which increases linearly with the number of measurements, i.e., it is of order $O(sN)$.

Next, we consider the higher-order correction terms. For $\frac{\partial l_k^{(G)}}{\partial\theta}$, which is the derivative of the Gaussian log-likelihood, it yields

$$\frac{\partial l_k^{(G)}}{\partial\theta} = \frac{\partial sNQ}{\partial\theta}(sNC)^{-1}(k-sNQ) - \frac{1}{2}(k-sNQ)\frac{\partial(sNC)^{-1}}{\partial\theta}(k-sNQ) - \frac{1}{2}\operatorname{tr}[(sNC)^{-1}\frac{\partial sNC}{\partial\theta}] \tag{S62}$$

Since $k - sNQ \sim \sqrt{sN}z$, the orders of the three terms above are $O(\sqrt{sN}z)$, $O(z^2)$, and $O(1)$, respectively.

For the term $\frac{\partial P_1}{\partial\theta}$, it can be expressed as

$$\frac{\partial P_1}{\partial\theta} \sim H_3(z)\frac{\partial\kappa}{\partial\theta} + \kappa\frac{\partial H_3(z)}{\partial z}\frac{\partial z}{\partial\theta}, \tag{S63}$$

and according to Eq. S55 with the order of $\frac{\partial z}{\partial\theta} \sim O(\sqrt{sN})$.

Substituting the above expression back into Eq. S61, the second term becomes

$$\frac{2}{\sqrt{sN}}E\left[\frac{\partial l_k^{(G)}}{\partial\theta}\frac{\partial P_1}{\partial\theta}\right] \sim \frac{1}{\sqrt{sN}}E\left[\left(O(\sqrt{sN}z) + O(z^2) + O(1)\right)\left(H_3(z) + \frac{\partial H_3(z)}{\partial z}\sqrt{sN}\right)\right]. \tag{S64}$$

Since the integrals of odd-order Hermite polynomials vanish under the Gaussian weight, and Hermite polynomials of different orders are mutually orthogonal, this term evaluates to zero.

For the third term,

$$\frac{1}{sN}E\left[\left(\frac{\partial P_1}{\partial\theta}\right)^2\right] \sim \frac{1}{sN}E\left[\left(H_3(z) + \frac{\partial H_3(z)}{\partial z}\sqrt{sN}\right)^2\right] \sim O(1). \tag{S65}$$

Therefore, for Eq. S61, the error term in the Fisher information obtained using the normal approximation is of order $O(1)$. Since the Fisher information itself is of order $O(N)$, the relative error is of order $O\left(\frac{1}{N}\right)$.

Figure S7 shows the standard deviation of the ToF estimates as a function of the number of measurements N, calculated via MC simulations under typical parameters. For comparison, the standard deviations obtained from the MC simulations at different N are normalized by the value at $N = 1000$, which is considered to approximate the large-N limit. It can be seen that for $N \geq 100$, the standard

deviations scale approximately as $1/\sqrt{N}$, as indicated by the dashed line in the figure. However, for $N < 100$, the deviations from this scaling become significant, indicating that the MLE is less likely to reach the theoretical limit in this regime

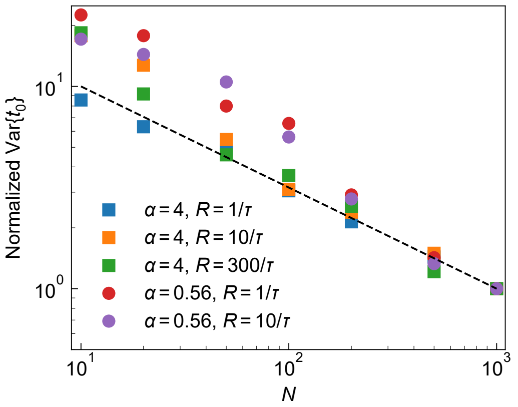

Figure S7: Comparison of normalized ranging standard deviations obtained via MLE (markers) with the theoretical performance bounds (black dashed line) for different numbers of measurements N, under varying pulse widths α and R for a Type I system with $s = 4$.

S7 Comparison with the Coates Algorithm

Here, the performance of the classical dead-time compensation method commonly known as the "Coates correction" is compared with the results obtained in this work [6]. The Coates algorithm is based on the following idea: according to Eq. (3) in the main text, the count in the i-th bin, h_i, follows a binomial distribution $B(N_i', q_i)$, where $N_i' = N - \sum_{j=1}^{T} hi - j$. For large N, and given the histogram, one can approximate $q_i \approx h_i/N_i'$, thereby removing the effect of the dead time as much as possible (since q_i depends only on the laser pulse shape, not on the dead time). Assuming a Gaussian laser pulse, the pulse center can then be estimated using the Center-of-Mass (CoM) method. Figure S8(a) illustrates the recovery of a dead-time-distorted histogram using the Coates algorithm. Compared with the theoretical photon-trigger probability distribution q_i, the raw histogram is shifted leftward due to pile-up effects. After applying the Coates correction, the recovered histogram shows much better alignment with q_i.

Figure S8(b) shows the ideal CRLB, the standard deviation of t_0 estimated via the MLE method proposed in this work, and the standard deviation obtained using the Coates + CoM method for different signal strengths R under typical parameters. It can be seen that the MLE-derived standard deviations closely match the theoretical CRLB across all R, whereas the Coates + CoM estimates consistently fail to reach the CRLB. Notably, when $R > 100$, the standard deviation from the Coates method may appear smaller than the CRLB; however, in this regime the pile-up effect is severe, and the t_0 estimated by the Coates method is no longer correct. These results demonstrate that the CRLB derived in this work provides not only the theoretical lower bound for ranging accuracy but also the ultimate performance limit for any unbiased dead-time compensation algorithm.

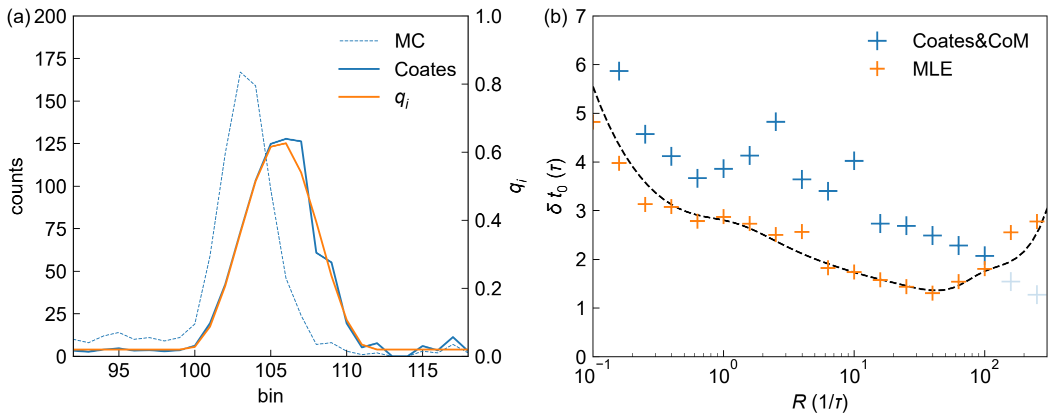

Figure S8: Effect of Coates Algorithm. (a) Comparison of original MC generated hist (blue dashed), hist compensated by the Coates algorithm (blud solid) and ideal q_i (orange solid). (b) Comparison of MLE method (orange cross), Coates+CoM method (blue cross) and theoretical performance bounds δt_0 (black dashed line) with varying R under $w = 4\tau$ and $t_0 = 100.1\tau$. The translucent blue markers indicate the cases with incorrectly estimated t_0.

S8 Performance Bounds for an Asymmetric Gaussian Pulse

As shown in the main text, the CRLB depends on the shape of the laser pulse. Since symmetric Gaussian pulses are widely adopted, our previous discussion focused primarily on this waveform. In practice, however, asymmetric Gaussian-like pulses are also common, such as those generated by RC discharge circuits. To investigate this case, we further examine the CRLB for an asymmetric Gaussian waveform. Specifically, we employed a Bézier curve to shift the center of a symmetric Gaussian pulse leftward by approximately 30%, as illustrated in Fig. S9.

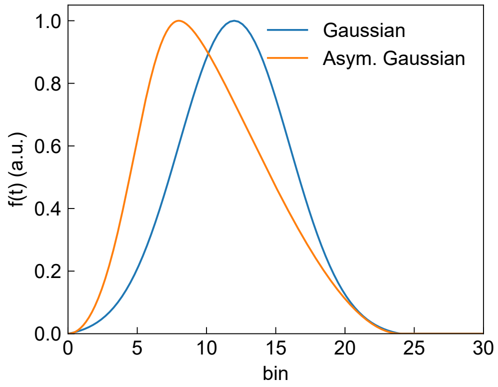

Figure S9: Comparison between a symmetric Gaussian pulse (blue) and an asymmetric Gaussian pulse (orange).

Figure S10 presents results analogous to Figs. 6(a) and 7(a) in the main text, showing the CRLB for the asymmetric Gaussian waveform under different pulse widths relative to the TDC resolution, along with the corresponding R_{opt}. Compared with the symmetric Gaussian case in the main text, the convergence behavior and order of magnitude of the CRLB remain essentially the same, while only the specific numerical values differ.

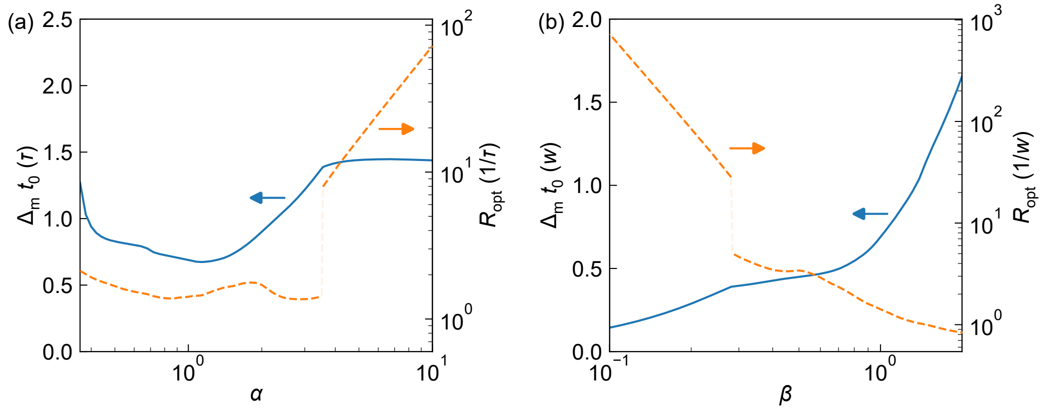

Figure S10: (a) Theoretical optimal ranging precision $\Delta_{\rm m} t_0$ (solid blue) and the corresponding optimal detection rate $R_{\rm opt}$ (dashed orange) for different $w = \alpha\tau$. (b) Theoretical optimal ranging precision $\Delta_{\rm m} t_0$ (solid blue) and the corresponding optimal photon rate $R_{\rm opt}$ (dashed orange) under varying $\tau = \beta w$. The translucent section of the $R_{\rm opt}$ curve indicates the region of abrupt transition.

S9 Sensitivity analysis of the theoretical ranging precision limit with respect to R

In the main text, it was noted that achieving the theoretical ranging precision requires operating the system at the optimal received photon flux R (i.e., $R_{\rm opt}$). This section analyzes the sensitivity of the ranging precision with respect to deviations from $R_{\rm opt}$.

As discussed in Section 3 of the main text, many figures illustrating the effect of varying R on system performance adopt a logarithmic axis for R. This implies that once the system is operating near $R_{\rm opt}$, its performance is relatively insensitive to small linear deviations in R. Figure S11(a) shows the relative error of theoretical ranging precision $\Delta_m t_0$ when R deviates from $R_{\rm opt}$ under different laser pulse widths at a fixed TDC resolution τ [corresponding to Fig. 6(a) in the main text]. Figure S11(b) presents the counterpart results for different TDC resolutions at a fixed pulse width w [corresponding to Fig. 6(b)]. As expected, even when R deviates from $R_{\rm opt}$ by as much as $\pm 20\%$, the resulting performance degradation remains at the order of only 1%.

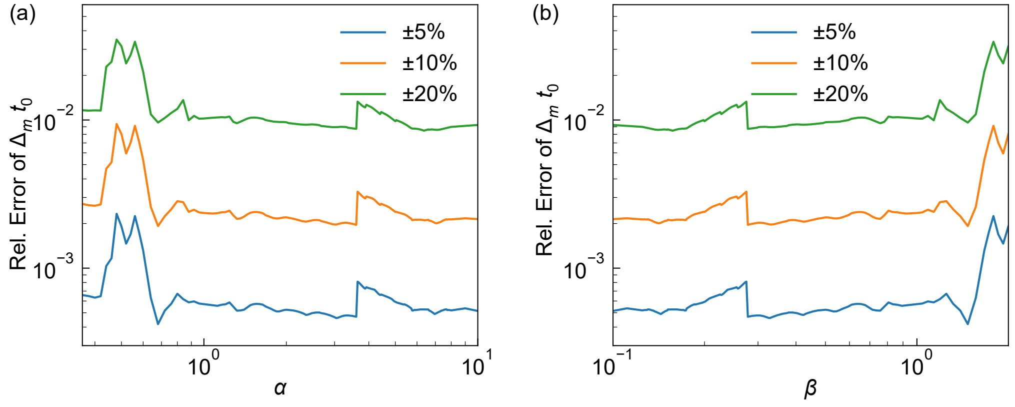

Figure S11: (a) Relative error of the theoretical optimal ranging precision $\Delta_{\rm m} t_0$ (solid blue) under different pulse widths $w = \alpha\tau$, when R deviates from $R_{\rm opt}$ by $\pm 5\%$ (blue), $\pm 10\%$ (orange), and $\pm 20\%$ (green). (b) Relative error of the theoretical optimal ranging precision $\Delta_{\rm m} t_0$ (solid blue) under different pulse widths $\tau = \beta w$, when R deviates from $R_{\rm opt}$ by $\pm 5\%$ (blue), $\pm 10\%$ (orange), and $\pm 20\%$ (green).

References

[1] Maciej Wojtkiewicz, Bruce Rae, and Robert K. Henderson. Review of Back-Side Illuminated 3-D-Stacked SPADs for Time-of-Flight and Single-Photon Imaging. *IEEE Transactions on Electron Devices*, 71(6):3470–3477, June 2024.

[2] Alfonso Incoronato, Mauro Locatelli, and Franco Zappa. Statistical Modelling of SPADs for Time-of-Flight LiDAR. *Sensors*, 21(13):4481, January 2021.

[3] Ivan Rech, Antonino Ingargiola, Roberto Spinelli, Ivan Labanca, Stefano Marangoni, Massimo Ghioni, and Sergio Cova. Optical crosstalk in single photon avalanche diode arrays: A new complete model. *Optics Express*, 16(12):8381–8394, June 2008.

[4] Yang Liu, Yihan Zhao, Jin Hu, Rui Ma, and Zhangming Zhu. An Efficient and Comprehensive Timing Jitter Model for Single Photon Avalanche Diodes. *IEEE Transactions on Electron Devices*, 71(10):6116–6123, October 2024.

[5] O. E. Barndorff-Nielsen and D. R. Cox. *Asymptotic Techniques for Use in Statistics*. Springer, London ; New York, 1989.

[6] P. B. Coates. The correction for photon ‘pile-up’ in the measurement of radiative lifetimes. *Journal of Physics E: Scientific Instruments*, 1(8):878, August 1968.
